# Relationship between Body Image and Body Weight Control in Overweight ≥55-Year-Old Adults: A Systematic Review

**DOI:** 10.3390/ijerph16091622

**Published:** 2019-05-09

**Authors:** Cristina Bouzas, Maria del Mar Bibiloni, Josep A. Tur

**Affiliations:** Research Group on Community Nutrition and Oxidative Stress, University of the Balearic Islands & CIBEROBN (Physiopathology of Obesity and Nutrition CB12/03/30038), E-07122 Palma de Mallorca, Spain; cristinabouvel@gmail.com (C.B.); mar.bibiloni@uib.es (M.d.M.B.)

**Keywords:** Body image, body weight, weight management, overweight, adults over 55 years

## Abstract

*Objective*: To assess the scientific evidence on the relationship between body image and body weight control in overweight ≥55-year-old adults. *Methods:* The literature search was conducted on MEDLINE database via PubMed, using terms related to body image, weight control and body composition. Inclusion criteria were scientific papers, written in English or Spanish, made on older adults. Exclusion criteria were eating and psychological disorders, low sample size, cancer, severe diseases, physiological disorders other than metabolic syndrome, and bariatric surgery. *Results:* Fifty-seven studies were included. Only thirteen were conducted exclusively among ≥55-year-old adults or performed analysis adjusted by age. Overweight perception was related to spontaneous weight management, which usually concerned dieting and exercising. More men than women showed over-perception of body image. Ethnics showed different satisfaction level with body weight. As age increases, conformism with body shape, as well as expectations concerning body weight decrease. Misperception and dissatisfaction with body weight are risk factors for participating in an unhealthy lifestyle and make it harder to follow a healthier lifestyle. Body image disturbance also made it more likely to underreport calorie intake. *Conclusions:* Aging is associated with a decrease in weight concerns and lower overweight perception, especially in women. However, when designing a program to improve body image in overweight ≥55-year-old adults, three items ought to be considered: physical activity, dietary and behavioral treatments.

## 1. Introduction

Obesity, and the state of being overweight, are conditions which are understood as an excess of body fat and are associated with increased risk of several diseases and direct causes of quality of life decrease, morbidity and mortality [1]. However, being overweight or obese is not necessarily enough be motivated to either start or continue weight management actions, since real body shape is sometimes misperceived [2]. Being overweight or obese could be perceived as normal weight. Less prevalent is the misperception of normal weight as overweight [3]. Hence, a correct self-perception of body image may affect body weight control and, eventually, quality of life. Moreover, body self-perception could be a helpful tool to health care providers when a body weight control program would be planned.

Body image defined as self-perception of weight, body shape or Body Mass Index (BMI) [4] might affect weight management. If actual self-perceived body image is far from ideal body image, it might initiate action to weight management [5]. This could be explained by the Higgins’ regulatory focus theory (RFT) [6] which refers to the relationship between a person’s motivation and pursuit of a goal. RFT postulates two different self-regulatory strategies: prevention focus goals (such as avoiding illness or social rejection) and promotion focus goals (such as desire to be attractive).

Obesity and the state of being overweight are stigmatized, and perception of adiposity was related to weight loss attempts in adults and children. It can be related to both psychological and physiological distresses, therefore having a negative impact on general health [3]. Body image has been widely studied in the young population, but it has been scarcely reported on in the aged population.

Otherwise, prevalence of non-transmissible chronic diseases increases after 55 years of age [7], and risk of suffering these diseases also increase with obesity or being overweight [7]. Hence the risk of diseases and comorbidities associated to an excess of weight increase with age [7]. Therefore, a better knowledge of body image and overweight perception in ≥55 years old people may contribute to more effective health practices, which might ultimately improve their health and quality of life [1]. To our knowledge, no systematic review has been made to date tackling body image and weight management strategies in the aged population.

This systematic review aims to assess the scientific evidence on the relationship between body image and body weight control in overweight ≥55-year-old adults.

## 2. Methods

The literature search was conducted using the Medlars Online International Literature (MEDLINE), via PubMed, and guided by the PRISMA statement [8]. The following search strategy was used: (Body Image [MeSH Major Topic]) OR (Body Image[Title/Abstract]) OR (Weight perception[Title/Abstract]) OR (Weight misperception [Title/Abstract]) AND (Body Composition [MeSH Term]) OR (Anthropometry [MeSH Term]) OR (Body Size [MeSH Term]) OR (Body Weight [MeSH Term]) OR (Weight [Title/Abstract])) AND ((Diet [MeSH Term]) OR (Diet [Title/Abstract]) OR (Diet Therapy [MeSH Term]) OR (Nutrition Therapy [MeSH Term]) OR (Diet Surveys [MeSH Term]) OR (Nutrition Surveys [MeSH Term]) OR (Diet, Food, and Nutrition [MeSH Term]) OR (Healthy Diet [MeSH Term]) OR (Food Preferences [MeSH Term]) OR (Food Preference[Title/Abstract]) OR (Food choice [Title/Abstract]) OR (Intake report [Title/Abstract]) NOT (Feeding and Eating Disorders [MeSH Terms]) NOT (Body Dysmorphic Disorders [MeSH Terms]).

The search was further restricted to studies published prior to December 2018. A total of 995 articles were identified. Then, three filters were applied: manuscripts published in scientific journals, written in English or Spanish, and studies including an ≥55-year-old adult population. Due to the very limited research on body image conducted exclusively on ≥55-year-old population, studies including our goal age range inside a broader age range were also included. We excluded articles where age was not specified (mean age or age range), and articles about the relation between eating and psychological disorders and preoccupations with appearance or self-image. As the review exclusively focused on psychologically and physically healthy population, articles including eating and psychological disorders, cancer, severe diseases, physiological disorders other than metabolic syndrome, and bariatric surgery, were excluded, as well as those with very low sample size (less than 20), population exclusively younger than 55 years, and articles not specifying age range or mean age. Literature reviews, expert opinions, editorials and reports were also excluded.

The articles were reviewed by at least two reviewers and were considered for the selection criteria listed on the Joanna Briggs Institute, a procedure to independently assess the methodological quality of scientific articles [9]. Quality in body image methodology was assessed using a review previously published [10]. Disagreement between reviewers was resolved by consensus.

**Figure 1:**
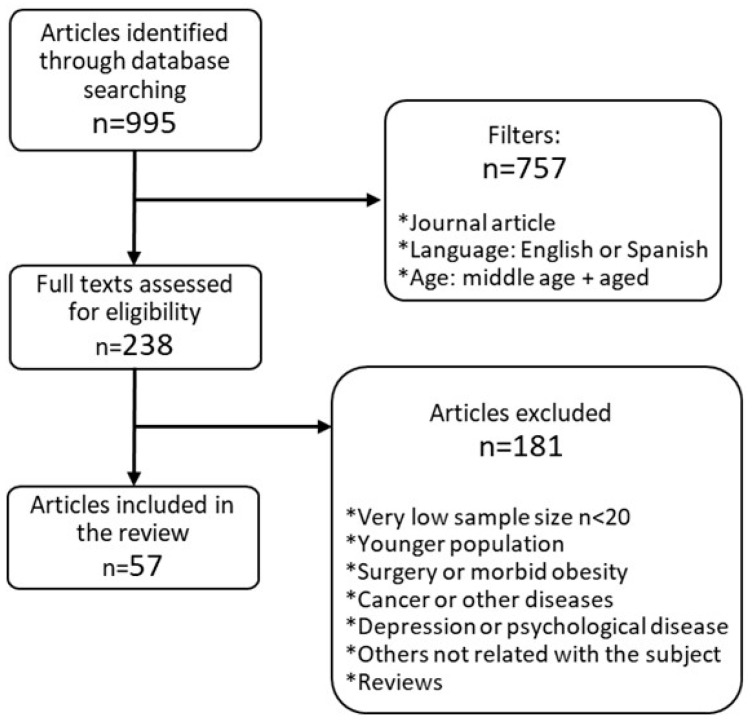
Flow-chart of study selection, inclusion and exclusion of studies.

Finally, fifty-seven articles were included in the review (Figure 1). To fulfil the objective of this review, they were grouped for the analysis into the following areas: (1) Body self-perception and weight management behaviors from a transversal point of view, regarding their relationship in adults in general, adults over 55 years, and comparison between genders, as well as the relationship between body image and food choices, diet quality and intake reporting. (2) Evolution of body image during weight loss management strategies.

## 3. Results

A total amount of fifty-seven studies were found. Of those, forty-nine were cross-sectional and eight were prospective. Most relevant results are shown in Table 1 and Table 2. Full contents of the results can be found in Tables S1 and S2.

Methods for body image analysis are not homogenous: some studies used a set of body silhouettes like those developed by Stunkard et al. [11], adapted to different ethnicities; others used validated questionnaires about weight perception or interviewed participants by asking body image related questions. Other studies combined methods.

Although the population studied often includes both genders, those concentrating on one gender only usually focus on women.

### 3.1. Body Self-Perception and Weight Management Behaviours from a Transversal Point Of View

The relation between body self-perception and diet-related behaviors is a hot topic for health care professionals. A total of 46 studies were suitable for this subheading. Forty studies tackled this subject in adult population although in six of them a secondary analysis by age groups was carried out. The remaining six focused on ≥55-year-old adults. Of the 40 studies including adult population, 29 considered both genders and 11 only considered women. The main tool for assessing body image were body image focused questions and less frequently, Stunkard’s silhouettes or validated questionnaires. Of the six studies conducted in ≥55-year-old adults, two included both men and women, one included exclusively men and three exclusively women. Body image was mainly assessed by Stunkard’s silhouettes and validated questionnaires.

Overweight self-perception in adults was associated with the wish to lose weight and pursuit weight control, regardless of real weight or gender [12,13,14,15,16,17,18,19,20]. Accordingly, overweight people misperceiving their weight were observed to be less likely to desire or pursuit weight control [12,17,21,22,23,24] than accurate weight perceivers. In very specific populations, such as senior Navajo Indians or non-educated Pakistani women living in Norway, heavier body shapes or heavier silhouettes were generally preferred [25,26]. On the other hand, in Europe and North America, normal weight or underweight perception in some cases led to weight-loss practices [24].

The main strategy for weight management was generally dieting, and less often, increasing physical activity or both [12,14,15,20,23,24,25,27,28]. Strategies for weight management varied between genders. While men preferred to exercise, eat less fat and look for advice from health professionals [15], women were more likely to join weight loss programs, follow a special diet [17,20], take pills, or eat more vegetables and fruits [28], although, at times, the diet they chose might be unhealthy or dictated by the trends. Participants in commercial weight loss programs were less satisfied with their health, appearance and body shape, and dieters compared to non-dieters had greater concerns about body image [16].

In general, body weight satisfaction was associated with less intention to change weight or lifestyles. On the contrary, body weight dissatisfaction, was associated with higher BMI and greater intention to change lifestyle or weight [29] and dietary restrain in women [30]. Another important motivation for weight management were concerns for either future or present health [20,31], especially in overweight and obese people [32] and in men, however for women, body image remained a concern regardless of body weight [33]. One Taiwanese study showed that women consider obese people to be clumsy, lazy, unhealthy and unattractive, regardless of their own weight status, however only obese women considered that obesity should be a hindrance for finding a job [34].

Weight satisfaction was associated with high self-reported ideal weight. For a given BMI, as ideal weight increases, so did the likelihood of weight satisfaction [29]. Nevertheless, usually, ideal weight falls within the normal weight range, being slightly lower in women in general [23], but higher in African American women [35]. Some overweight and obese African American women also perceived themselves as attractive [20]. This was more relevant when Latino or Caucasian women were compared to African American women [36]. Ideal weight or weight loss expectations when starting a weight management program related to the maximum weight loss achieved in previous weight loss attempts [31] and was lower than the body silhouette chosen as realistic shape [37]. Ideal BMI was not affected by history of dieting in women [38]. However, weight loss expectations were not always predicted by weight dissatisfaction [31].

Healthy lifestyles were related to normal weight perceptions, especially among men [39]. On the other hand, body image dissatisfaction was associated with either low rates of physical activity or a reduction over time in physical activity, high energy intake, or a great reduction of intake through time [29,40]. Overweight men perceiving themselves as overweight were less likely to meet the exercise recommendations [41]. As a matter of fact, exercise enjoyment seemed to have a small effect in reducing body image concerns in women losing weight [42]. Moreover, even when health was similar, non-dieters felt healthier than dieters. However, since dieters were enrolled in what to them seems a healthy lifestyle, they felt fitter than non-dieters [16].

When genders were taken into account, women were more likely than men to perceive themselves as overweight or obese and more worried about their weight, hence more likely to control their weight [15,43,44]. Moreover, women’s weight losses perceived as reasonable or realistic (assessed by body silhouettes) were greater than men’s [37]. If height is considered, being tall was more important for men than for women [45]. When women had to identify attractive female silhouette for men, they choose a thinner silhouette than the one chosen by men as attractive female. Women think that men like thinner women than they actually do, as opposite to men, who agreed with women in the male attractive silhouette [37]. Regarding shape preferences in women only, although they could easily identify if their actual shape was pear or apple, their preferred silhouette was predominantly pear [46].

Few studies tackle the issue of body image and weight management exclusively in the population over 55 years or adjust their analysis by age groups. Compared to general adult population, less people over 65 years old had an overweight perception were currently trying to lose weight or were increasing their physical activity, however those differences were reduced when analyzing adults between 45–64 years [24]. In African American middle-aged diabetics, overweight men and women perceived themselves to be heavier than they would like and acting to control body weight was related to both body size perception and satisfaction [47], as was for general adult population. Concerns about body size in middle age influence the report of not only weight and height, but also waist circumference measures. BMI was associated to mis-reporting waist circumference for both genders, however only females trying to control their weight were more likely to underestimate their BMI [48]. Men over 55 years and women under 55 years were more likely to desire to lose weight than younger men and older women respectively; yet only younger women were more likely to be acting to control their weight than any other group [9]. When comparing overweight and obese older women with younger women, the older group settled for lower weight loss expectations, however body satisfaction was similar among age groups [49]. Compared to younger, women, women over 40 years old were more likely to self-perceive themselves as overweight, to have ever dieted and to have lost at least 5 kg during their lifetime [32]. Considering this exclusively women over 50 years, aging was associated to a decrease in weight concerns [50].

The Charleston Heart Study included two parallel studies conducted in ≥66-year-old women and ≥55-year-old men respectively and aimed to compare ethnicities. Caucasian men and women were more likely than African Americans to have ever dieted, to report lower ideal than perceived weight, BMI or silhouette, and also to perceive themselves to have a bigger silhouette than they had, regardless of their current weight status. In African American men weight control practices were closely related to education while in women there were no differences between weight categories [36,51]. African American women tended to be satisfied with their weight and silhouette and consider themselves attractive [36]. On the other hand, Caucasian women were satisfied and felt attractive only if normal weight [36]. Generally, men felt attractive regardless of their body image [51].

Dietary patterns were directly associated with weight management and health, which were both related to body weight and, ultimately, to body self-perception. Body weight satisfaction was associated with healthy lifestyles (healthy diet and exercise), but less intention to change weight or diet. On the contrary, weight dissatisfaction was associated with higher BMI and less healthy lifestyles (low exercise and poor diet), but with greater intention to change lifestyle [29]. In women, an unhealthy dietary pattern consisting of high intakes of carbohydrates, sweet drinks and refined foods was associated with obesity, large body image or body silhouette and high lifetime silhouette increase, while a healthy pattern rich in vegetables, fruits and cereals was associated with a low BMI and silhouette and the smallest change in silhouette and BMI over time in women [52]. As already mentioned, strategies for weight management vary between genders. While men preferred to eat less fat, women were more likely to eat more vegetables and fruits [28]. Obese women also tended to avoid visible fat from food; however, their diet was rich in high-fat food and sugary beverages [34]. Overweight perception among overweight women was related to a higher fruit intake while overweight perception among overweight men was associated with less fruit intake [41]. In this regard, obese adults, especially those with overweight perception, were willing to pay more for healthier snacks [53]. Overweight or obese self-perception in women was also related to incomplete Mediterranean meals, as opposed to normal weight perception [54].

Regarding to age, a population with a mean age of 78 years were more likely to eat complete Mediterranean meals, combining carbohydrates, proteins, vegetables and fruits [54]. Accordingly, in women aged 50–75 years, living in the US, weight or appearance concerns were related to healthier dietary patterns, such as high fish, chicken and fiber consumption, as well as low percentage of total caloric intake from fat [50].

Lower ideal than reported weight was associated with higher perceived importance of nutritional facts in adults living in the US [55]. Desire to weigh less increased the likelihood of choosing low calorie foods [55]. In another study conducted in Hispanic descendent adults living in the US, meeting the exercise guidelines and overweight perception were associated with ordering foods with less calories [56]. However, weight perception was not associated with the frequent use of nutrition facts, at least before the new regulation [55].

Relation between body image or weight status satisfaction and energy intake reporting is complex. In studies conducted in adults, it was found that body shape or body weight dissatisfactions were related to low energy reporting in 24 h diet recalls. Adults considering themselves overweight, adults trying to lose weight at the time of the interview or during the past year, regardless of their current weight, adults with lower desired than real weight or weight gainers in the past 10 years were more likely to underreport energy intake by 304, 398, 339 and 17 kilocalories per kg gained, respectively [57,58]. Generally, in women aged 50–75 years, body image discordance was not associated with protein or energy underreporting [59].

### 3.2. Evolution of Body Image During Weight Loss Management Strategies

Fourteen studies were included in this subheading. Six were observational and eight interventional. Among the observational, one was longitudinal, conducted in women over 50 years and used Stunkard’s silhouettes. The remaining five were conducted in adults and used single related questions or validated questionnaires to assess body image. None of the latter conducted any secondary analysis stratified by age. All the interventional studies were conducted in adults and no secondary analysis stratifying by age was carried out. Body image was assessed by validated questionnaires, computer-created pictures or using Stunkard’s silhouettes. Four were conducted in both genders, while the rest were conducted exclusively in women.

Accuracy in estimating body image increased after the end of a specific diet or a weight loss program [18]; however, with time, participants tended to underreport their body weight [60].

In most studies, body image improved after weight loss [16,44,61,62,63,64,65]; however, dieters were as vulnerable to body image relapse as they were to regain weight [64]. Women’s body image could also improve only by increasing exercise, regardless of the weight change [61]. In a randomized study conducted in obese women, participants were encouraged to be physically active and attend weekly sessions about either meal replacement or a hypocaloric low-fat diet or healthy eating. Body image concerns were greatly reduced, regardless of weight loss, and associated with less internalization of body image standards [66]. Two studies compared the effect of weight loss alone and body image psychological therapy (with [64] or without weight loss therapy [65]) on body image. One was conducted on both genders [64] while the other exclusively in women [65]. In the first study there were no differences between groups regarding weight loss [64]. In the second study, the weight loss group lost more weight than the psychological therapy group; however, differences were lost after one year [65]. For what body image and body image satisfaction are concerned, they both improved regardless of the intervention [64,65]. A lower concern for body image was a predictor of long-term weight loss when there was no intervention regarding body image concerns [62].

One study conducted in women living in Australia found that a personalized future self-image (computer-designed image of the volunteer in the future, if the same lifestyle continues) boosted weight loss efforts. This motivational tool is more effective at the middle of the programme [67].

During a weight loss program, individuals with an early onset of overweight states or obesity did not decrease their body image concerns as quickly as those with a late onset, indicating that body image concerns were more persistent in early onset individuals [44]. An observational study conducted in ≥50-year-old women, showed that dissatisfaction with body image had a direct relation with lifetime weight change cycles [40]. Body image dissatisfaction was associated to either low or a reduction over time in physical activity, high energy intake, or a great reduction of intake through time [40].

Big weight loss expectations can have a negative effect on actual weight loss [68]. In fact, attrition rates were proportionally related to baseline weight loss expectations [68].

## 4. Discussion

The most consistent evidence to emerge from this review was that, in the western world, perception of being overweight or obese, as well as body weight dissatisfaction or low ideal BMI were associated to some sort of weight management [12,13,14,15,16,17,18,19,20,21,22,23,24]. This is within the common sense. Bearing in mind the amount of evidence and consistency of the different sources, the authors concluded that weight perception has influence on weight management. In fact, the use of external assistance for weight control was mainly motivated by psychological aspects of obesity, instead of health concerns [69]. In this regard, a particular study conducted in Australia should be mentioned. They identified overweight people’s ulterior motive for starting a weight loss program. Health was the main motive, then body image, and the less common was mood [70]. Those findings are aligned with some other studies included in this review [20,31,32]. This is closely related to the finding that, when health is similar and regardless of the weight management method used, dieters felt less healthy than non-dieters, nevertheless they felt fitter [16]. It seems coherent that health perception is lower in dieters than in non-dieters as health concerns were an important motivator for starting a weight loss program, yet former and current dieters tend to be heavier than non-dieters, which could be influencing health [71]. Despite this, body image satisfaction alone was related to health. For a given BMI, body image satisfaction was associated to both a healthy lifestyle and general health. However, these findings might be explained by a selection bias [72]. Likewise, in Danish general population from 20–59 years overweight perception was associated to lower vitality and general health, regardless of the current weight; however, only for women was real weight interfering in their quality of life [33]. Speaking of quality of life, people ongoing a weight loss treatment had a higher perceived quality of life than weight loss maintainers [42]. This could partially be explained by the excitement and the expectations of the weight management itself, which could boost optimism.

Weight management strategies usually consisted of dieting and sometimes exercising or combining both [12,14,15,20,23,24,25,27,32]. It was described that the more dissatisfied a woman is with her own body size, the more likely it is for her to use an undesirable weight control method [19,34]. This should be carefully considered by health practitioners, to prevent eating disorders and health damages. However, unhealthy weight management strategies were rarely used in the evidence included. A recent review tackling the use of either healthy or unhealthy weight loss strategies in general population, including adolescents, could not provide a clear answer about the likelihood of choosing healthy over unhealthy or vice versa. Therefore, feeling overweight does not relate with the healthiness of the weight management strategy chosen to handle weight [3].

For both genders, appearance was significant; however, appearance was more important for women [15,43,44]. Women preferred a pear body shape [45], while for men, one of the key issues was their height [45]. In men, height was associated with desirable looks and success. Hence men dissatisfied with their height were more likely to also be dissatisfied with body shape and weight, which led men to weight control strategies, since weight is a parameter that they can control as opposed to height [45]. Despite that, only women failed in trying to assess an attractive female figure for men, while men and women agreed in the male attractive figure [37]. The pressures that women experience regarding their appearance should not be forgotten. On one hand, the media promotes thinness and beauty. Media pressure proved to increase the likelihood of wanting to lose weight [19]. On the other hand, social and food environments promote high calorie consumption and undermine weight loss efforts [73]. Accordingly, women might experience body shame and anxiety, which could ultimately lead to social isolation, simply because they avoid any social event involving food, physical activity or light clothes [71]. Back to the topic of weight concerns, there is an exception that seems to be consistent through literature: African American women had less concerns about their weight. They may identify themselves as overweight but still feel attractive, therefore they might not be trying to control their weight [20,36]. This pressure does not only influence the preferred body shape, but also the weight management strategy used and the expected weight loss after a management strategy. Accordingly, in general population, and more strongly among obese people, self-reported ideal weight has increased over time [29].

It is also interesting that low physical activity at the enrolment of a weight management program or a decrease of physical activity through time led to body image dissatisfaction [40]. In fact, in the same study where women’s preference for a pear shape was described, they also proposed exercise as a strategy to switch from an apple to a pear shape as opposite to diet, which does not change or modulates shape [45]. Therefore, body image could be a personal motivation for senior women to start exercising [74]. Furthermore, physical activity modified body perception. Compared with being sedentary, practicing physical activity helped to have an accurate body perception [75]. In a different study, body image dissatisfaction was associated with lower levels of physical activity for both men and women, as opposed to body image satisfaction, associated with greater odds to practice moderate or vigorous activities [76]. Nevertheless, it should be considered that some BMIs that would classify as overweight are not caused by an excess of body fat, but a great muscle mass that can be due to an intense exercise program. This is more likely to happen in men than women [42]. In aged adults, weight control, including physical activity, could influence mental health by maintaining a satisfactory body image [77]. Hence, the relation between physical activity and body image may be bidirectional.

Unhealthy lifestyles were associated with both obesity and greater body image [28,29,39], however aged people were more likely than younger members of the society to eat complete and healthier meals [50,54]. However, in this study conducted in Italy, these results may be explained because aged Italians were attached to the Mediterranean diet [54]. It was found that both lower ideal than reported weight as well as overweight perception were associated with higher concern about calories in foods [55,56]. Food and nutrition education has a crucial role to play, since food companies tend to take advantage of this “calorie-content” interest to promote their products, which may not always be the healthier choice. On the other hand, besides controlling calorie intake, while the general adult population overweight perception or weight management were related to energy intake underreporting [57,58], in aged women, either overweight, obesity or body image concerns were not related to energy or protein underreporting [59]. This is crucial when assessing someone’s intake prior to starting a weight management plan or a weight-related intervention.

Whenever the focus is set on aged people, some differences were found among genders and ethnicities. Caucasians were more likely to perceive themselves to be more overweight than they were [36,51]. For African Americans, body size satisfaction is the trigger to start a weight management action [47]. Evidence exposed in this review regarding the fact that older women were as concerned as younger women about their body shape and their attitudes towards it; however, aged women resigned to lower weight loss expectations [49,50] which is consistent with existing reviews on this topic [71]. Older men were less likely than younger men to be satisfied with their body and therefore they were more likely to restrain eating [51]. There was one particular study conducted in middle aged women that came to an opposite conclusion than those conducted in adults. In this study they saw that aged women trying to control their weight were more likely to underestimate their BMI [48]. However, evidence on people over 55 years is lacking, therefore more research on this population is needed.

Evidence regarding the evolution of body image during weight loss management strategies showed that after a weight loss, body image improves [16,44,61,62,63,64,65]. However, in women, evidence claims that this is not so straightforward. Body image in women improved not only due to weight loss, but also due to behavioral treatment or physical activity [40,42,61,65,66]. Furthermore, behavioral interventions improved body image more than dietary interventions [78]. This might be related to the fact that often, diets are related to feelings of guilt if “forbidden” foods are consumed, which makes eating unenjoyable and unsuccessful dieters get trapped in a vicious circle damaging mental health [71]. Emotional approaches to weight loss treatments could be a tool to avoid this vicious circle. It should also be kept in mind that improvements in body image led to greater satisfaction with the weight management program, as was shown in a study in which participants tended to feel more satisfied with the program as their body image perception improved, regardless of their body weight [79].

In the general population, and more strongly among obese people, self-reported ideal weight has increased over time [29]. If weight loss expectations are not realistic, they could be a hindrance for actual weight loss [68], since they were related to higher expected speed of weight loss in women18. After weight loss, self-estimations of body image became more accurate, nevertheless, when time passes body weight tended to be underestimated [18,60].

### Strengths and Limitations

A strength of the present review is that it includes not only observational studies, but also interventional studies. This allowed examination of not only motivations and relation among body image and the different outcomes, but also its evolution during weight management. Another strength derives from the fact that it includes all the evidence available in this subject, since there was no exclusion criteria regarding year of publication. Finally, body image is crucial when a weight management program is designed. Body image not only has an impact at the beginning of weight management, determining the perceived importance and willing to engage in such program, but also impacts in the satisfaction with and the adherence to the program. Furthermore, an accurate body image in the long term predicts not only dietary adequacy preventing malnutrition, so prevalent among the older population, but also socially and healthy lifestyles, especially among women.

Nonetheless, this review has some limitations. Publication bias could be the first limitation influencing the present review. As a matter of fact, most of the included studies are conducted in the most developed countries. This makes the results only applicable in those cultures. Secondly, very few studies tackle body image exclusively in population over 55 years old or perform analysis adjusting by age, and the majority only considered women. More studies are needed in this age group, including men in their sample. There are several studies conducted among adults over 20 years old, which include also population over 55 years, however, in most of those studies all subjects have been analyzed together. Most of them did not perform any secondary analysis in every age category. It is important to highlight that in most of studies conducted in adults, the upper age range limit was set on 65 years and only includes the younger individuals of the aged population. More studies in people over 55 years old, of both genders, would be needed to allow arrival at a stronger conclusion. Thirdly, methodology was not homogenous through bibliography. The most used method consisted of some body image-related single questions. Questions such as “what is your current weight?”, “what would be your ideal weight?” or “do you consider yourself normal-weight, overweight or obese?” were the main tool used in about 40% of the articles included. This percentage rises when the focus is set only on observational studies. This may be due to the fact that most surveys were performed over the telephone, and it is a very simple method that can be easily performed over the telephone. Silhouettes and validated questionnaires regarding body image were the main tool used in around 30% of the articles. This variety of methodology was kept in mind through this present review, as stated in the methodology. Finally, the review fails to take into consideration body image according to the different theoretical paradigms (psychoanalysis, behavioral and cognitive theories). The search did not apply any filter regarding theoretical paradigms, as the authors decided to simply focus on the relationship between body image in general and body weight control in overweight ≥55-year-old adults.

## 5. Conclusions

Overweight self-perception is related to spontaneous weight management in overweight adults. Moreover, body image concerns improve as body weight reduces. Weight control strategies should take into account not only dietary treatment, but also behavioral treatment and physical activity, as they all contribute to improve body image.

In ≥55-year-old overweight and obese population, evidence is very limited because studies generally include broad age ranges. This could affect results, diluting the actual results of ≥55-year-old people. In the few studies carried out in our target population, findings suggest that aging is associated with a decrease in weight concerns and lower overweight perception. Nevertheless, when weight perception in overweight people is accurate, this is associated to weight control, as for general adults.

Yet there are differences between genders in ≥55-year-old population. Men over 55 years are more likely than younger ones to desire to lose weight, as opposed to women of the same age. Younger women are more likely to desire a weight loss than ≥55-year-old women. Although women seem to be less concerned with their weight as they grow older, they still tend to find themselves unattractive if they are overweight. This is not the case for African American women, who tend to be more satisfied with their image than Caucasians.

Lifestyle affects body image satisfaction for both general adult population and ≥55-year-old women. There is no available evidence in this regard on ≥55-year-old men. In ≥55-year-old women, low physical activity, high energy intake or a big reduction in energy intake are associated with body image dissatisfaction.

Despite the limitations of both lifestyle and behavioral interventions, clinical trials are needed to provide stronger evidence in this issue and population.

## Figures and Tables

**Table 1:** Observational studies.

Ref	Objective	Sample	Main Results
**54**	Association between meal type choice and weight perception accuracy.	- Italy - N = 516 - 3 age groups	**Age** - Young adults (OR = 4.0), senior men (OR = 2.80) and senior women (OR = 2.59) were the most likely to eat complete meals. **Weight perception:** - NW perception was related to complete meals (OR = 2.76 M; 2.54 W). - OW perception in women was related to incomplete meals (OR = 4.12).
**53**	Body image influence on consumers’ willingness to pay for potato chips carrying nutritional claims	- Spain - N = 309 - Age unspecified	**Willingness to pay for chips with nutritional claims** (low-salt or low-fat): - NW with OW perception > OB with OW perception. - Body image dissatisfaction of NW people did not influence the consumers’ willingness to pay.
**55**	Associations of weight self-perception with food choice intentions and consumer response to calorie information.	- USA - N = 639 low income adults. - Aged 15–75	**Food choice intentions:** - Desire to weigh less (OR = 2.0) increased the likelihood of choosing low calorie food. **Calorie information use:** - Desire to weigh less increased the perceived importance of calorie information in grocery stores and fast-food and chain restaurants. - Self-perception of weight was not associated with the frequency of use of calorie information.
**57**	Association between low energy intake reporting and weight management and self-perception.	- NHANES 2007–2012 - USA - N = 13.581 - Age > 20 years	**Weight perception:** - Low energy reporters: OW: 66.0%; NW: 31.0%; UW: 3.0%. - Incorrect OW perception increased the likelihood of intake underreporting (OR = 1.54). **Weight management** (tried to lose weight in the past year): - Wanting to weigh less (OR = 1.29), weight attempts in the past year (OR = 1.56) and desire to weigh less (OR = 2.51) increased the likelihood of intake underreporting.
**56**	Effect of menu labels on calories and macronutrients ordered from specific foods.	- USA - N = 372 Hispanic descents - Aged 18–65	**Ordering fewer calories was significantly associated to:** - Overweight perception (β = −124.4). - Meeting the exercise guidelines (β = −202.3).
**28**	Gender differences in weight-related outcomes across BMI spectrum.	- NHANES (2009–2010) - USA - N = 4258 OW or OB - Aged > 20	**Weight management:** - Men attempting to lose weight were more likely to eat less fat (OR = 1.23) and exercise (OR = 1.45) than women. - Men were less likely to join a weight loss program (OR = 0.16), take prescription diet pills (OR = 0.17), follow a special diet (OR = 0.58), and eat more fruits, vegetables, and salads (OR = 0.73) than women.
**40**	Factors contributing to dissatisfaction with body image.	- Women’s Health Initiative Observational Study (1993–2002) - USA - N = 75.256 women - Aged: 50–79	**Body image dissatisfaction was associated with:** - At baseline: lower physical activity or higher energy intake. - At 3 years: a decrease in physical activity or a greater reduction in energy intake.
**12**	Body image in relation to body mass index and weight control.	- SANHANES-1 (2012) - South Africa - N = 6411 - Aged >15	**Perceptions:** - Wanted to be thinner: 23.4%. Wanted to be larger: 21.9%. **Weight management:** - Attempted to lose weight: 12.1% (mainly those who perceived themselves as larger). Diet: 62.4%; physical activity: 38.7%; supplements: 9.2%; others: 9.2%. - Attempted to gain weight: 10.1% (mainly those who perceived themselves as thinner). Diet: 85.6%; physical activity: 6.8%; supplements: 9.3%.
**49**	Comparison of young and old women on their weight loss expectations and related attitudes.	- Italy - N = 26 women (aged 18–38) + 33 women (aged 60–78)	**Weight loss expectations:** - Older: 11.7–24.0%. Younger: 18.3–31.5%. **Body dissatisfaction:** - Did not differ between younger and older women.
**39**	Factors associated with weight misperception.	- DANSDA (1995, 2000–2004 and 2005–2008) - Denmark - N = 9623 - Aged: 15–75	**Lifestyle:** - Weight misperception increased with higher levels of leisure-time physical activity. - Intention to eat healthy (OW men): ○ Usually trying and never trying→ weight misperception. ○ Occasionally trying → accurate weight perception. **General health** (Good health perception decreases odds of perceiving overweight in): - OW men: 2.73 times; OW women: 2.28 times.
**62**	Long term weight loss factors.	- Italy - N = 88 OW or OB - Aged 18–65	**Body weight and body image:** - Shape concern correlated with percentage of body weight change 10 years after treatment (r = 0.36). - No other predictors of 10-year body weight change.
**42**	Test exercise in the weight loss maintainers.	- Portugal - N = 321 women - Aged 18–65	**Physical activity:** - Weight losers: exercise enjoyment slightly reduced body shape concerns. **Body image.** - Body shape concerns and body image dissatisfaction: weight loss treatment > weight loss maintainers > not attempting weight loss. - Perceived hunger: weight loss treatment > weight loss maintainers.
**59**	Psychosocial and diet behavior factors affecting dietary self-report.	- WHI-NPAAS study - USA - N = 450 women. - Aged 50–79	Body image discordancy was not significantly associated to energy or protein underreporting.
**52**	Relationship between self-perceived body shape, BMI and dietary patterns.	- EsMaestra (2006–2008) - Mexico - N = 18.875 OW or OB female teachers. - Aged 37–77	**Association between BMI and dietary pattern:** - Vegetable pattern: lower actual BMI (OR = 0.77); lower life increase (OR = 0.79). - Carbohydrate pattern: higher actual BMI (OR = 1.47); higher life increase (OR = 1.27). **Association between silhouette and dietary pattern:** - Vegetable pattern: lower actual silhouette (OR = 0.68); lower life increase (OR = 0.76). - Carbohydrate pattern: higher actual silhouette (OR = 1.86); higher life increase (OR = 1.56).
**21**	Associations between weight misperception and weight-related attitudes and behaviors.	- NHANES (2003–2006) - USA - N = 20.470 (BMI >25) - Aged >20	**Weight management** Overweight misperceivers compared to accurate perceivers: - Want to lose weight: 71% M; 65% W. - Not attempted to lose weight the past year: 60%M; 56%W. - Physical activity: 32.0% men less likely to be insufficiently active.
**13**	Association between perceived overweight and weight control. Discrepancies between perceived and measured weight.	- NHANES (2003–2004 + 2005–2006 + 2007–2008) - USA - N = 16.720 - Aged > 18	**Desire to lose weight:** - Age group: <55 y-o: 53.7% M and 74.9% W. >55 y-o: 59.0% M and 69.5% W. - Perception: NW: 48.2% W. OW: 95.2% M and 98.3% W. - Health care professional diagnosis of excessive weight: 91.4% M and 93.9% W. **Pursuit of weight control:** - Overall: 48.4% (39.6% M and 57.1% W). Overweight perception: 59.6% M (OR 3.74); 71.6% W (OR 2.82). - Age group: <55 y-o: 39.2% M and 60.5% W. >55 y-o: 40.7% M and 50.1% W. - Health care professional diagnosis of excess weight: 65.0% M; 75.1% W.
**26**	Body size perceptions in a RCT to prevent deterioration of glucose tolerance with diet and physical activity.	- Norway (Pakistani women living in Oslo) - N = 198 OW or OB women - Aged: 25–62	- Actual perceived body size: 5.7. **Discrepancy score:** (self-perceived—preferred by Pakistani living in Norway) - Positive: 79%. (higher percentage in higher BMI groups). - Negative among BMI 25.0–27.5: 20%.
**22**	Relationship between weight perception and weight management behaviors.	- NHANES (1999–2006) - USA - N = 11.319 OW or OB - Aged > 20	**Weight management:** - Misperceivers: ○ Tried to lose weight 29.1%. Tried not to gain weight 29.7%. ○ Desired weight: less 33.3%; same 63.3%. - Accurate perceivers: ○ Tried to lose weight 61.1%. Tried not to gain weight 52.5%. ○ Desired weight: less 97.3%; same 2.5%.
**30**	Effect of eating on body image satisfaction.	- UK - N = 46 women. - Mean age: 36,7 ± 12,9	**Mean body image satisfaction correlated to:** - BMI (r = −0.47). - Dietary restraint (r = −0.65).
**29**	Relationship between ideal weight perception, weight satisfaction and health practices (weight cycling, physical activity, and diet)	- Aerobics Center Longitudinal Study (1997 + 2001) - USA - N = 19,347 - Aged 20–87	**Body weight satisfaction:** - BMI was associated with a lower odds of weight satisfaction (OR: 0.49 M; 0.47 W) and intentions to change weight (OR: 1.85 M; 2.10 W). - Higher ideal weight was associated with a higher odds of body satisfaction (OR: 1.07 M; 1.09 W) and less intention to lose weight (OR: 0.95 M; 0.94 W). **Physical activity and energy intake:** - Weight satisfaction was associated with higher physical activity, higher fruits and vegetables intake, higher cardiorespiratory fitness and lower intention to change diet. - Intention to change diet: 93% men and 95% women dissatisfied with their weight. - Lower fruit and vegetable consumption: OW men dissatisfied with their weight and women (regardless of the BMI) dissatisfied with their weight. OW men dissatisfied with their weight consumed less fruits and vegetables.
**14**	Association of weight perception and participation (baseline) in an intervention promoting weight control through lifestyle changes.	- USA - N = 899 employees of hospitals - Aged 18–65	**Weight management:** - Currently trying to lose weight: 62.5%. (45% NW; 65% OW; 78% OB). ○ Diet and or Physical activity: 43.7% (29.0% M; 47.6% W); bariatric surgery: 2%. **Perceived weight status and weight loss attempts** - Slightly OW perception: Men: OR = 14.4; women: OR = 8.9. - Moderately OW perception: Men: OR = 13.8; women: OR = 8.2. - Very OW perception: Men: OR = 9.6; women: OR = 5.0.
**41**	Prevalence of meeting recommended levels of leisure-time physical activity and fruit and vegetable consumption. Effects of weight status and perceptions.	- Australian National Health Survey (2004–2005) - Australia - N = 16,314 - Aged > 20	**≥ 2 servings fruit/day:** - OW: NW perception: 50.7%M; 61.9%W. OW perception: 46.1%M; 63.4%W. ○ OW itself (OR: 1.11) and OW perception in OW (OR: 1.14) increased the likelihood of having ≥ 2 servings fruit/day for women. ○ OW perception in OW men (OR: 0.83) decreased the likelihood of having ≥ 2 servings fruit/day - OB: NW perception:52.8 %M; 59.5%W. OW perception: 45.2%M; 62.4%W. **Physically active:** - Excess weight (except for OW men) and OW perception were related to less exercise.
**34**	Body image, attitudes toward overweight people, and dietary behaviors.	- Taiwan - N = 96 women - Aged: 20–59	**Attitudes towards obesity** (%agreement) - Employers should not hire overweight people. 56.5% NW. 76.0% OB. - Overweight people are unattractive. 78.3% NW. 94.0% OB. - All had a negative attitude towards obese people. **Dietary behaviors** - OB group showed less control over portion size, fried foods and sugar-containing beverages.
**38**	Associations between weight expectations and anthropometric profile.	- Canada - N = 154 women - Mean age 42.4 ± 5.6 (BMI 25–35)	**Diet and weight history:** - Similar: age of first weight loss attempt, number of times dieting, highest and lowest weight during adult life. **Satisfaction with their weight:** Realistic ideal BMI > unrealistic ideal BMI.
**19**	Impact of BMI, advertising and media on the nutrition transition and their impact on eating styles and body image.	- Jordan - N = 800 women - Aged 18–73	**Desire to lose weight:** - Media pressure: increases 2.908 times the likelihood of wanting to lose weight. - Compared to NW: ○ OW increases 11.14 times the likelihood of wanting to lose weight. ○ OB increases 18.05 times the likelihood of wanting to lose weight.
**27**	Perception of weight, attempts to lose weight.	- BEACH program - Australia - N = 1973 - Aged > 18	**Weight management strategies** (% use): - Diet or exercise: 56.6% OB; 40.0% OW; 20.0% NW. - General practitioner advice: 26.2%OB; 11.7% OW; 2.4% NW.
**68**	Influence of weight loss expectations on attrition.	- QUOVADIS study - Italy - N = 1785 OB - Aged 25–65	**Attrition predictors:** - Expected 1 year loss (6 months: HR = 1.19; 12 months: HR = 1.12). Attrition risk increase 12% per 1 kg/m^2^ desired loss. - Body image (6 and 12 months: HR = 1.42). **BMI loss predictors:** Baseline BMI (β = 0.28); age (β = −0.16); age at first dieting (β = −0.10). Body image was not a predictor.
**15**	Weight perception and management practices.	- USA - N = 198 OB Latinos (Community men and women; and Labor camp) - Aged 18–64	**Perceived overweight:** - Community: 68.6% M; 83.3% W; Labor camp: 73.7% M. **Weight management** (% currently trying to lose weight): - Community: 55.7% M; 72.2% W; Labor camp: 26.3% M. - Among overweight perceivers (% currently trying to lose weight): Community: 67% M; 75% W; Labor camp: 36%M. **Weight management strategies** (% use): - Dieting: Community: 84.6% M; 93.8% W; Labor camp: 80.0% M. - Exercising: Community: 61.5% M; 52.3% W; Labor camp: 50.0% M. - Health care provider advice: Community: 59% M; 80% W; Labor camp: 37% M.
**43**	To assess self-perception of body weight, attitudes toward weight-control behaviors, and associated factors.	- Spain - N = 1200 - Aged: 20–65	**Overweight self-consideration** (Do you consider yourself overweight or obese?): - UW or NW: 1.8% M; 16.4% W. - OW or OB: 52.0% M; 84.0% W. **Weight management** (ever dieted): - UW or NW: 20.0% M; 48.0% W. - OW or OB: 46.0% M; 84.7% W.
**31**	Weight loss expectations in obese patients seeking treatment.	- QUOVADIS - Italy - N = 1891 OB. - Aged 25–65	**BMI loss expectations:** (mean kg/m^2^; *p* < 0.001 between sexes) - Expected 1-year loss: related to maximum previous lost (r_s_ = 0.30; *p* < 0.0001). **Weight loss motivation** (primary): - 15.2% Appearance (7.4% M; 17.4% W). - 51.5% Present Health (56.0% M; 50.2% W). - 33.4% Future Health (36.6% M; 32.5% W).
**35**	Potential correlates of obesity (dietary intake, body image, and physical activity).	- Caretakers of children in the Hip-Hop to Health Jr. (5-year RCT) - USA - N = 305 women - Aged 18–67	**Body image** (figure rating scales) (*p* < 0.01 between ethnicities): - Ideal Body image: Black: 3.54 ± 0.07; Hispanics: 3.14 ± 0.07.
**48**	Levels of dietary restraint are associated with mis-reporting measures of adiposity.	- Parents of participants in Barry Caerphilly Growth Study - UK - N = 227 - Middle-aged	- BMI was associated with the mis-reporting of waist circumference for both genders. **Dietary restraint score** - Women: +1 Dietary restraint score = + 0.36 kg/m^2^ self-reported—measured BMI.
**58**	Characteristics of misreporting of energy intake during 24-hour dietary recalls.	- USA - N = 98 - Aged 25–73	**Weight perception:** - −304 kcal underreported for considering oneself overweight or desire to weigh less. **Weight management** (tried to lose weight in the past year): - −339 kcal underreported for desire to weigh less. - −398 kcal underreported for having attempted weight loss. - −17 kcal underreported per kg gained in the past 10 years.
**23**	Weight perceptions and weight control are associated with body weight.	- Omnibus Survey of the Office of National Statistics (1999) - UK - N = 1799 - Age >16	**Ideal weight** (In the range of normal weight. Higher among men) **Weight control:** - Correlations to trying to lose weight: BMI: men: r = 0.42; women: r = 0.48 Perceived BMI: men: r = 0.52; women: r = 0.56. **Methods:** Diet: 26.2% M; 51.5% W. Advice from professional: 3.0% M; 4.4% W.
**46**	Erroneous expectations about the likely outcomes of weight loss diets.	- N = 62 women - Mean age: 33 ± 9	- Both groups were accurate selecting current body shape. - Both groups (androgynous and pear-shaped) chose the pear-shaped image as diet outcome.
**33**	Dieting history, measured and perceived weight.	- MORGEN (1989) - Netherlands - N = 4601 - Aged 20–59	**Weight perception:** - Men: 5.9% UW perception; 54.8% NW perception; 39.3% OW perception. - Women: 3.1% UW perception; 51.8% NW perception; 45.1% OW perception.
**50**	Attitudes about weight, appearance and food and nutrient intakes.	- USA - N = 221 women - Aged 50–75	- Age was inversely related to weight concerns (r = −0.22) **Food intake and body image:** - Weight concerns were associated to higher fish and chicken consumption (r = 0.23) and the association to a higher fiber intake was suggested. - Appearance concerns were associated to lower % of energy as fat (r = −0.13).
**32**	Weight status perception, accuracy of self-assessment of weight status and weight control practices relative to the degree of adiposity.	- MORGEN project (1995) - Netherlands - N = 4601 dieters - Aged 26–65	**Weight perception:** - Women over 40 y-o were less likely to self-perceive OW or OB than younger (OR = 0.7). **Weight control motivations:** OW or OB individuals: health. NW individuals: appearance.
**37**	Realistic shape perceptions, current and ideal or attractive shape and weight perceptions.	- USA - N = 20000 dieters (3 years after diet) - Mean age: 45 ± 15.	**Weight perception** (significant differences between men and women): - Ideal shape < realistic shape < current shape (for all). Ideal and realistic: lower for women. - Reasonable weight loss: higher than ideal weight (8.7% M < 14.7% W). - Men: accurate male attractive figure. Women: inaccurate in female attractive figure.
**25**	Weight, body image, and weight control practices	- Navajo Health and Nutrition Survey - Navajo Indians (USA) - N = 786 - Aged >20	**Weight perception:** - Ideal weight of OW: 20–59 y-o: 14% M; 7% W. > 60 y-o: 31% M; 17% W. **Current weight control:** - Age: 20–39 y-o: 43% M; 59% W. 40–59 y-o: 38% M; 43% W. > 60 y-o: 10% M; 29% W. **Methods:** Diet: 82%; pills: 3%, other products: 3%; vomiting after eating: 4%.
**47**	Body image perceptions and attempts to alter weight.	- USA - N = 370 diabetic African Americans - Mean age: 50 ± 13	**Weight perception:** - Correlation perceived size and BMI: Men r = 0.77; Women: r = 0.76. - Desired own size ∓ current: OW > NW; trying to change weight>not trying; bigger for women. **Current weight control:** - Perceived size correlated to weight control in OW (Men r = 0.3; Women r = 0.2). - Body size satisfaction was negatively related to weight control.
**60**	Accuracy of self-reported weight.	- Baseline from a worksite health promotion (1988–1991) - USA - N = 4432 - Mean age: 38 ± 10	**Weight control related to weight underreporting:** - Following a weight loss program was related to weight underreporting. - History of weight loss was related to weight underreporting.
**36**	Attitudes toward eating and body size perceptions	- Charleston Heart Study (1991) - USA - N = 517 women - Aged 66–105	**Weight perception:** - Perceived current silhouette: white > black. OW > NW. - Discrepancy score (ideal-perceived weight): Black: 0.3; White: 1.4; NW: 0.3; OW: 1.6. - Ideal BMI – perceived BMI: Black: 1.0; White: 2.8; NW: 0.2; OW: 4.7. **Weight management** (differences in weight categories): - Feeling attractive: White: 67% NW; 37% OW. No differences in black women (71.5%). - Ever tried to lose weight: White: 49% NW; 86% OW. No differences in black (52.5%).
**51**	Examine body size perceptions, dieting and cognitive control of intake in different ethnicities.	- Charleston Heart Study (1991) - USA - N = 334 men - Aged 55–98	**Weight perception:** - Perceived current silhouette: white > black. OW > NW. - Discrepancy score (ideal-perceived weight): Black: 0.4; White: 0.9; NW: 0.2; OW: 1.5. - Ideal BMI – perceived BMI: Black: 0.7; White: 1.3 NW: -0.2; OW: 2.9. **Weight management** (differences in weight categories): - 78% feel attractive. - Ever tried to lose weight: 37% Black (42% NW; 33% OW). 64% White (49% NW; 82% OW). Differences in weight categories and ethnic groups. (Black: affected by education).
**16**	Diet enrollees’ initial body image compared to controls	- “New Direction” by Cash + Ross Laboratories (1991) - USA - N = 360 - Age: 41.4 ±10.3	**Weight satisfaction:** - Dieters reported less satisfaction with their appearance and body. **Health:** - Dieters felt less healthy than controls (objective health was similar for both cohorts). - Dieters felt fitter than controls. - Dieters were more conscious about their appearance and fitness.
**24**	Knowledge and practices regarding weight loss, trends between 1985–90.	- NHIS - USA - N = 55545 - Aged >25	**Weight perception:** - OW: 36.7% M; 52.0% W. 25–44 y-o: 43.8%; 45–64 y-o: 52.4%; > 65 y-o: 35.7%. - UW: 6.2% M; 4.5% W. 25–44 y-o: 5.1%; 45–64 y-o: 3.7%; > 65 y-o: 8.6%. **Weight management** (trying to lose weight): - 23% M; 40%W. 25–44 y-o: 35.5%; 45–64 y-o: 34.7%; > 65 y-o: 19.7%. - Weight perception: OW: 58.6%; NW: ~12% (1985 > 1990). UW: ~ 2% (1985: < 1990). **Weight management strategies** (% use): - Methods (from best to worst): Eat few calories > not eating before going to bed > physical activity > others. If combining: few calories + physical activity. - Eating less: 76.4% M; 83%W. 25–44 y-o: 78.9%; > 45 y-o: 82.5%. - Increasing activity: 60% M; 57.5% W. 25–44 y-o: 64.5%; 45–64 y-o: 53%; > 65 y-o: 40.5%.
**20**	Relevant factors to design and implement weight control programs.	- USA - N = 500 black women - Aged 25–64	**Weight perception:** - Weight satisfaction: OB: 3%; OW: 5%; NW: 19%; UW: 47%. - Feeling attractive: OB: 39%; OW: 44%; NW: 74%; UW: 85%. Predictor: younger age. **Weight management strategies:** - Currently trying: OB: 37%; OW: 31%; NW: 25%; UW: 14%. - Ever tried: OB: 90%; OW: 88%; NW: 78%; UW: 41%. Motive: health and appearance. - Strategies: Diet 39% (in UW: 28%); Exercise: 12%; Diet + exercise: 45%.
**45**	Relationship between height and body image parameters related to dieting, body weight and shape.	- Canada - N = 174 - Mean age: 40 ± 15.7	**Body shape concerns:** - Positively correlated to BMI: Men (r = 0.39). Women (r = 0.51). - Negatively correlated to high in men (r = −0.25) (independent of body weight). **Weight management:** - Positively correlated to BMI in women (r = 0.27). - Negatively correlated to high in men (r = −0.25) (independent of body weight).
**17**	Risk behaviors of the overweight compared with average weight. Association between body image, weight status, and dieting.	- BRF 1981–1983 - USA - N = 19405 - Aged 18–79	**Weight perception:** - Accurate weight perception: OW women > OW men. White > black. **Weight management:** - Dieting is related to weight status (28% OW Men; 48% OW Women). - More females than males are dieting within each weight group. - OW perception is related to dieting. - The proportion of overweight on a diet increases with perception of weight status.

Abbreviations: BMI: Body Mass Index. UW: Underweight, NW: Normal weight, OW: Overweight, OB: Obese. M: Man, W: Women. y-o: years-old.

**Table 2:** Intervention studies.

Ref	Objective	Sample	Intervention	Main Results
**67**	Effect of a personalized future self-image on weight change over a 6-month period.	- Australia - N = 121 OW or OB - Aged: 18–79	- Time of watching future self-image (image of the volunteer in the future, if the same lifestyle continues) + late new image (random): At baseline + new image. At baseline + No new image. 8 weeks delayed + new image. 8 weeks delayed + No new image. - All received 15 min of lifestyle advice. Participants chose weight management method.	**Weight loss:** - Week 16: delayed-image: −0.50%; early-image: −0.30%. - Week 24: delayed-image: −0.50%; early-image: −0.27%. - Greater weight loss for the delayed-image group. - Late (second) image did not influence weight.
**61**	Best treatment. Association between body satisfaction and physical activity.	- USA - N = 107 physically inactive OB women. - Aged 30–65	- Treatment 24 weeks. All encouraged to increase physical activity (150 min/week). Randomized to: ○ Coach Approach exercise-support (group sessions). ○ LEARN (low fat) Program for Weight Management (written manual + phone support).	**Body satisfaction predicted body weight change:** - 3 month satisfaction predicted 6 month weight (β = −0.35). - 6 month satisfaction predicted 12 month weight (β = −0.51). - 12 month satisfaction predicted 24 month weight (β = −0.41). **Body weight change predicted body satisfaction:** - 3 month weight predicted 6 month satisfaction (β = −0.34). - 6 month weight predicted 12 month satisfaction (β = −0.42). - 12 month weight predicted 24 month satisfaction (β = −0.47). **Physical activity:** Changes in physical activity related to body satisfaction, even after controlling for weight change and treatment.
**63**	Develop and test the effectiveness of the program.	- USA - N = 90 OW or OB - Aged 20–72	- 6 months (20 weekly sessions). Diabetes Prevention Program (Behaviors modification, healthy eating and physical activity). - Continuing care (continue meeting for 18 months following treatment). - Control (no advice or dissuasion to continue meeting).	**Body image** - Significant effect for time. Body image improves in continuing care group. (f = 8.30).
**66**	Changes in obesity-related attitudes.	- USA - N = 123 OB women - Mean age: 44.2 ±10.0	- Weekly group sessions. All encouraged to increase physical activity (180 min/week). Randomized: ○ Diet therapy: meal replacement. ○ Diet therapy: balanced hypocaloric self-selected diet. ○ Non-dieting program (eating healthy).	**Body image improvement** (Improvement through time): - Dieting group: week 20: 18.4%: week 40: 22.0%. - Non-dieting group: week 20: 15.9%: week 40: 26.2%. - No differences between groups. **Body image correlated with:** - Self-esteem (week 20: r = 0.23; week 40: r = 0.36). - Less internalization of society’s appearance ideals (week 40: r = 0.35).
**44**	Body image disturbance before and after weight loss.	- USA - N = 82 OW or OB - Aged 18–60	- Diet: medically supervised liquid formula given freely (4 weeks). - Mean weight loss of 6.4± 3 kg.	**Distortion (actual shape):** M > W. Caucasians >African American - After weight loss: Early onset >adult onset (f = 10.2). **Discrepancy (ideal shape):** M > W. Caucasians > Hispanics - After weight loss: Early onset >adult onset (f = 8.5). **Body image disturbance after weight loss** - Decreased significantly for all. Early onset >adult onset.
**64**	Compare weight management programs.	- USA - N = 65 OW or OB - Aged 19–63	2 randomized intervention (16 weeks): ○ Weight control (LEARN = low fat). 1 h/week dietitian. ○ Weight control + body image (basic calorie control + body image psychological therapy). 1 h/week dietitian + 2 h psychologist.	**Body image in both groups:** Improved through time (severe to normal range). **Body image in weight control (without body image therapy):** - Body image improvement through treatment correlated to greater weight loss (r = 0.53) and weight loss retention (r = 0.42). **Body image dissatisfaction predictors:** - Weight regain (r = 0.29 and r = 0.31). - Low weight loss retention (r = 0.56).
**65**	Evaluate a treatment for weight management.	- UK - N = 75 OW or OB women - Age: 18–65	Group intervention (10 week): healthy diet + physical activity + focus on: ○ Standard (control): energy restriction (1200 kcal). ○ Modified (intervention): self-control + self-esteem. No focus on weight loss.	**Weight loss:** - After treatment: standard: 3.9 kg (t = 5.92): modified: 1.3 kg. - After 1 year: standard: 3.6 kg (t = 2.32): modified: 2.0 kg (t = 2.41). (f = 3.71). - Significant differences (Standard > modified) at the end of the treatment, but not after 1 year. **Body image:** No group by time interactions. Improvements through time in body dissatisfaction (f = 12.44).
**18**	Body preoccupation as an indicator of distortions in body image.	- Australia - N = 68 women - Aged 18–65	- Weight reduction counselling courses at Macquarie University (slow lifestyle modification). - Once a week: initial session + 10 clinical sessions (intensive counselling) + 4 measurement sessions (2^nd^6^th^11^th^15^th^contact).	- ideal > goal > actual - All saw themselves more obese than they were. **Actual perceived body image** (% weight over perceived): - Baseline: Completers (19%) < drop-outs (26%) (t = 7.8). - Baseline: Low BMI (19%) < high BMI (24%) (t = 5.03). - Baseline (19%) > 15 week (8.8%) (real 8.9% less weight).

Abbreviations: BMI: Body Mass Index. UW: Underweight, NW: Normal weight, OW: Overweight, OB: Obese. M: Man, W: Women. y-o: years-old.

## Supplementary Materials

The following are available online at https://www.mdpi.com/1660-4601/16/9/1622/s1, Table S1: Observational studies; Table S2: Intervention studies.
