# Supplementary material for: Relationship between Body Image and Body Weight Control in Overweight ≥55-Year-Old Adults: A Systematic Review
Source: Int J Environ Res Public Health. 2019 May 9;16(9):1622. doi: 10.3390/ijerph16091622 (PMC6540116; doi:10.3390/ijerph16091622)
Supplement: Supplementary file 1 [file ijerph-16-01622-s001.pdf]

International Journal of
Environmental Research and Public Health

MDPI

Review

Relationship between Body Image and Body Weight Control in Overweight ≥55-Year-Old Adults: A Systematic Review

Cristina Bouzas, Maria del Mar Bibiloni and Josep A. Tur *

Research Group on Community Nutrition and Oxidative Stress, University of the Balearic Islands & CIBEROBN (Physiopathology of Obesity and Nutrition CB12/03/30038), E-07122 Palma de Mallorca, Spain.

* Correspondence: pep.tur@uib.es; Tel: 34-971-1731; Fax: 34-971-173184

Abstract: *Objective*: To assess the scientific evidence on the relationship between body image and body weight control in overweight ≥55-year-old adults. *Methods:* The literature search was conducted on MEDLINE database via PubMed, using terms related to body image, weight control and body composition. Inclusion criteria were scientific papers, written in English or Spanish, made on older adults. Exclusion criteria were eating and psychological disorders, low sample size, cancer, severe diseases, physiological disorders other than metabolic syndrome, and bariatric surgery. *Results:* Fifty-seven studies were included. Only thirteen were conducted exclusively among ≥55-year-old adults or performed analysis adjusted by age. Overweight perception was related to spontaneous weight management, which usually concerned dieting and exercising. More men than women showed over-perception of body image. Ethnics showed different satisfaction level with body weight. As age increases, conformism with body shape, as well as expectations concerning body weight decrease. Misperception and dissatisfaction with body weight are risk factors for participating in an unhealthy lifestyle and make it harder to follow a healthier lifestyle. Body image disturbance also made it more likely to underreport calorie intake. *Conclusions:* Aging is associated with a decrease in weight concerns and lower overweight perception, especially in women. However, when designing a program to improve body image in overweight ≥55-year-old adults, three items ought to be considered: physical activity, dietary and behavioral treatments.

Keywords*:* Body image; body weight; weight management; overweight, adults over 55 years.

Table S1. Observational studies.

Ref	Objective	Sample	Evaluation methods	Results	Limitations
**60**	Association between meal type choice and weight perception accuracy.	– Italy<br>– N=516 (258 men + 258 women): 156 children (mean age 10y-o), 187 young adults (mean age 24y-o), 173 seniors (mean age 78y-o)	– Measured weight and height<br>**Body image:**<br>– Perceived weight (that led to estimated weight accuracy)<br>**Other:**<br>– Food-Based Classification of Eating Episodes	– Complete meal: carbohydrate source + protein source + vegetables and fruits.<br>**Age**<br>– Young adults (OR=4.0), senior men (OR=2.80) and senior women (OR=2.59) were the most likely to eat complete meals.<br>**BMI**<br>– NW males (OR=9.33) and females (OR=3.58), and OW males (OR=2.67) were the most likely to eat complete meals.<br>**Weight perception:**<br>– NW perception was related to complete meals (OR=2.76 M; 2.54 W)<br>– OW perception in women was related to incomplete meals (OR=4.12)<br>– Accurate weight perception was related to complete meals (OR=2.33 M; 3.91 W).<br>– Overestimating weight was related to lower complete meals (OR=0.38) in men.	– Includes young people and kids in the same analysis<br>– Age range unspecified<br>– Eating disorders may be included in the analysis.
**60**	Body image influence on consumers' willingness to pay for potato chips carrying nutritional claims	– Spain<br>– N=309<br>– Age unspecified	– 12 choice tasks: three alternatives (two different images of chips carrying several nutritional claims and prices, and a no-purchase scenario). Participants had to purchase and pay for the potato chips they picked.<br>**Body image:**<br>– Body Image State Scale	**Willingness to pay for chips with nutritional claims** (low-salt or low-fat)**:**<br>– OB with OW perception > OB with NW perception.<br>– NW with OW perception > OB with OW perception.<br>– OB with NW perception > NW with NW perception.<br>– Body image dissatisfaction of NW people did not influence the consumers' willingness to pay.	– Age unspecified<br>– Study sponsored by a food company
**60**	Associations of weight self-perception with food choice intentions and consumer response to	– USA<br>– N=639 low income adults.<br>– Aged 15-75	**Body image:**<br>– Actual and ideal weight<br>– Body weight discrepancy (current - ideal weight)<br>**Calorie information**<br>– Questions about the use of calorie information postings	**Food choice intentions:**<br>– Desire to weigh less (OR=2.0) increased the likelihood of choosing low calorie food.<br>– Desire to weigh more (OR=0.5) decreased the likelihood of choosing low calorie food.<br>**Calorie information use:**<br>– Desire to weigh less (OR=1.5) and desire to weight more (OR=1.2) increased likelihood to think that calorie information should be posted. (nor significant)	– Includes young people<br>– Data obtained before regulation of nutritional facts in labels.

	calorie information.			– Desire to weigh less increased the perceived importance of calorie information in grocery stores and fast-food and chain restaurants.<br>– Desire to weigh less was not related to the use of calorie information on food packages when purchasing them the first time.<br>– Self-perception of weight was not associated with the frequency of use of calorie information.	
**60**	Association between low energy intake reporting and weight management and self-perception.	– NHANES 2007-12<br>– USA<br>– N=13.581<br>– Age > 20 years	– Measured weight and height.<br>**Body image:**<br>– **C**onsidered themselves overweight, underweight, or average<br>– Desired to weigh different.<br>– Tried to lose weight in the past 12 months<br>**Physical activity and energy intake:**<br>– Two 24h diet recall<br>– Goldberg cut-off method for energy reporting (intake / metabolic rate)<br>Global Physical Activity Questionnaire	**Weight perception:**<br>– Low energy reporters: OW: 66.0%; NW: 31.0%; UW: 3.0%.<br>– Non-low energy reporters: OW: 49.5%; NW: 45.7%; UW: 4.8%.<br>– Incorrect OW perception increased the likelihood of intake underreporting (OR=1.54).<br>**Weight management** (tried to lose weight in the past year)**:**<br>– Low energy reporters: 25.1% succeed, 29.0% failed and 45.9% did not try.<br>– Non-low energy reporters: 19.2% succeed, 20.2% failed and 60.6% did not try.<br>– Wanting to weigh less (OR= 1.29), weight attempts in the past year (OR=1.56) and desire to weigh less (OR=2.51) increased the likelihood of intake underreporting.	– Includes young people<br>– No measured weight
**60**	Effect of menu labels on calories and macronutrients ordered from specific foods.	– USA<br>– N=372 Hispanic descents (127 no labels, 123 energy needs, 122 calorie labels)<br>– Aged 18-65	– Random assignment to menus with:<br>o no labels (n=127)<br>o rank-ordered calorie labels and energy needs per meal (n=123)<br>o rank-ordered exercise needed to consume calories labels (n=122)<br><br>– Self-reported height and measured weight<br>**Body image:** (Not specified)<br>– Overweight self-perception.	– Noticing or using the labels did not associate to ordering less calories<br>– Men ordered more calories than women.<br>**Ordering more calories was significantly associated to:**<br>– BMI (β11.4).<br>**Ordering fewer calories was significantly associated to:**<br>– Overweight perception (β=-124.4)<br>– Meeting the exercise guidelines (β=-202.3)<br>– Choosing restaurant foods based on health value (β=-249.9)	– Includes young people<br>– Self-reported height<br>– Does not specify techniques to assess body image
**60**	Gender differences in weight-related outcomes	– NHANES (2009-10)<br>– USA	– Measured height and weight.<br>**Body image:**<br>– Self-perception of overweight, underweight, or normal weight.	**Body image:**<br>– Men were less likely than women to have accurate weight perception (OR = 0.31), weight dissatisfaction (OR = 0.33).<br>**Weight management:**	– Includes young people

	across BMI spectrum.	– N= 4258 overweight or obese (2125 women + 2133 men)<br>– Aged > 20	**Weight management:**<br>– Desire to lose weight.<br>Weight loss attempts in the past year, and if it was successful.	– Men were less likely than women to have attempted weight loss (OR = 0.53),but those attempting men were more likely to have successful weight loss (OR = 1.51).<br>– Men attempting to lose weight were more likely to eat less fat (OR = 1.23) and exercise (OR = 1.45) than women.<br>– Men were less likely to join a weight loss program (OR = 0.16), take prescription diet pills (OR = 0.17), follow a special diet (OR = 0.58), and eat more fruits, vegetables, and salads (OR =0.73) than women.	
**60**	Factors contributing to dissatisfaction with body image.	– Women's Health Initiative Observational Study (1993-2002)<br>– USA<br>– N= 75.256 postmenopausal women<br>– Aged: 50-79	Data collection: Baseline + 3 years<br>– Anthropometric measurements.<br>**Body image**:<br>– Figure rating scales (actual, ideal → body image dissatisfaction score)<br>**Weight management:** weight/dietary history before baseline (history of weight change).<br>**Physical activity and energy intake:**<br>– Energy expenditure from recreational physical activity.<br>– WHI food frequency questionnaire.	– Mean body image discrepancy score was 1.27± 1.03 (dissatisfied)<br>**BMI fluctuation:**<br>– Increasing +1 kg/m²: +0.0813 in body image dissatisfaction score (↑ dissatisfaction)<br>– Difference 3-years - baseline: +1 kg/m²: +0.0753 in body image dissatisfaction score (↑ dissatisfaction)<br>**Body weight satisfaction** (rates of reporting weight satisfaction in the conditions)**:**<br>– Stable adult weight: 32.4%<br>– Weight lost and maintenance as an adult: 37.5%<br>– Steady gain in weight: 6.3%<br>– Weight cycling as an adult: 8.5%<br>**Body image dissatisfaction was associated with:**<br>– At baseline: lower physical activity or higher energy intake.<br>– At 3-years: a decrease in physical activity or a greater reduction in energy intake.	– Only post-menopausal women.
**60**	Body image in relation to body mass index and weight control.	– SANHANES-1 (2012)<br>– South Africa<br>– N= 6411<br>– Aged >15	– Measured height and weight<br>**Body image**:<br>– Figure rating scales (actual, ideal for Body Image Distortion and Feel Ideal Difference indexes respectively)<br>**Weight management:** attempts lose/gain weight	**BMI prevalences:**<br>– Overweight: 50.8%; normal-weight: 42.1%; underweight: 7%.<br>**Perceptions:**<br>– Overestimated their BMI: 35.0%.<br>– Wanted to be thinner: 23.4%. Wanted to be larger: 21.9%.<br>**Weight management:**<br>– Attempted to lose weight: 12.1% (mainly those who perceived themselves as fatter). Diet: 62.4%; physical activity: 38.7%; supplements: 9.2%; others: 9.2%.<br>– Attempted to gain weight: 10.1% (mainly those who perceived themselves as thinner). Diet: 85.6%; physical activity: 6.8%; supplements: 9.3%.	– Includes young people and **adolescents** in the same analysis.
**60**	Comparison of young and old women on their weight loss	– Italy<br>– N= 26 young women (aged 18-38) + 33	Data collection just before weight loss treatment.<br>**Body image**:<br>– Created and validated questionnaire about weight goals	– Older women reported larger perceived and ideal figures than younger women.<br>**Weight loss expectations:**<br>– Older: 11.7 - 24.0%. Younger: 18.3 – 31.5%<br>– Higher BMIs were associated with higher weight loss expectations.<br>**Body dissatisfaction:**	– Only women<br>– No longitudinal study (Cross-

	expectations and related attitudes.	older women (aged 60-78).	– Body dissatisfaction (subscale Eating Disorders Inventory)<br>– Body Image Assessment for Obesity	– Did not differ between younger and older women.<br>– Did not correlate with weight loss expectations at any age.	sectional study)<br>– Comparison between age groups.
**60**	Prevalence of overweight and weight misperception.<br><br>Factors associated with weight misperception.	– DANSDA (1995, 2000-04 and 2005-08)<br>– Denmark<br>– N= 9623 (randomized sampling)<br>– Aged: 15–75	– Self-reported weight and height.<br>– Lifestyle and self-rated health<br>**Body image:**<br>Self-perception of overweight status	**Prevalence of overweight:** increased from 1995 to 2008<br>**Weight misperception:**<br>– OW men: 1995: 77.5 %; 2008: 71.4 %.<br>– OW women: 1995: 54.8 %; 2008: 51.9 % (not statistically significant)<br>**Lifestyle:**<br>– Weight misperception increased with higher levels of leisure-time physical activity.<br>– Intention to eat healthy (OW men)<br>o Usually trying and never trying→ weight misperception<br>o Occasionally trying → accurate weight perception<br>**General health**(Good health perception decreases odds of perceiving overweight in)**:**<br>OW men: 2.73 times; OW women: 2.28 times	– Includes young people.<br>– Self-reported weight and height.
**60**	Psychological and behavioural issues before and 10 years after a nutritional treatment.<br><br>Long term weight loss factors.	– Italy<br>– N=88<br>– Aged 18-65 overweight or obese	– Measured weight and height.<br>**Body image:**<br>Prior to weight-management program:<br>– Dieting Readiness Test<br><br>At 10-year follow-up:<br>– Eating Disorder Examination Questionnaire	– Weight loss success: decrease over 0,5kg. Failure: increase over 0,5kg.<br>**Dieting**<br>– No differences on self-reported treatments (prescribed or self-prescribed) of hypocaloric diets.<br>**Body weight and body image:**<br>– Shape concern correlated with percentage of body weight change 10 years after treatment (r = 0.36).<br>– No other predictors of 10-year body weight change	– Includes young people<br>– Includes morbid obesses<br>– Different questionnaires at baseline and 10-year follow up.<br>– Weight loss treatment unspecified
**60**	Compare quality of life, psychological well-being, and eating self-regulation	– Portugal<br>– N= 321 women (107 trying to lose 5Kg, 107 weight loss	**Body image:**<br>– Body Image Assessment questionnaire<br>– The Body Shape questionnaire<br>**Physical activity and diet related:**	**Sample description:**<br>– Weight loss maintainers: lost 17% of initial body weight. Kept weight off for 2.5years.<br>– Treatment: lost 8% of initial body weight at the end of the behavioural program.<br>**Physical activity:**<br>– Weight losers: exercise enjoyment reduced, a little, body shape concerns.	– Only women<br>– Secondary objective.<br>– Includes young people

	among groups.<br><br>Test exercise in the weight loss maintainers.	maintainers, 107 not trying to lose weight)<br>– Aged 18-65	– Minutes of moderate or vigorous physical activity per week (METs)<br>– Intrinsic Motivation Inventory (adapted)<br>– The Eating Inventory	**Body image.**<br>– Body shape concerns and body image dissatisfaction: weight loss treatment > weight loss maintainers > not attempting weight loss.<br>– Perceived hunger: weight loss treatment > weight loss maintainers	
**60**	Psychosocial and diet behaviour factors affecting dietary self-report.	– WHI-NPAAS study<br>– USA<br>– N=450 women.<br>– Aged 50-79	**Body image:**<br>– Figure rating scales (Stunkard) (actual, ideal)<br>**Energy intake:**<br>– Dietary assessments (FFQ, four day food record, 24h diet recall)<br>– Energy intake: double labelled water<br>– Protein Intake: urinary nitrogen	Body image discordancy was not significantly associated to energy or protein underreporting.	– Only women<br>– Techniques validating energy intake were not evaluating the same days as 24h diet recalls or four day food record
**60**	Relationship between self-perceived body shape, BMI and dietary patterns.	– EsMaestra (2006-08)<br>– Mexico<br>– N= 18.875 female teachers.<br>– Aged 37-77<br>– BMI> 25 (*self-measured)	– Self-measuredheight, hip, waist and weight.<br>**Body image:**<br>– Stunkard’s Figure Rating Scale (2 years after menarche, between 18 - 20 y-o, before first pregnancy, between 25 - 35 y-o, actual)<br>**Physical activity and energy intake:**<br>– 116-item FFQ<br>Physical activity → MET-h/week	**Dietary patterns:**<br>– Vegetable pattern: high intakes of fruit, vegetables, nuts and cereals<br>– Carbohydrate pattern: high intakes of carbohydrates, sweet drinks and refined foods<br>**Association between BMI and dietary pattern:**<br>– Vegetable: lower actual BMI (OR=0.77); lower life increase (OR=0.79)<br>– Carbohydrate: higher actual BMI (OR=1.47); higher life increase (OR=1.27)<br>**Association between silhouette and dietary pattern:**<br>– Vegetable: lower actual silhouette (OR=0.68); lower life increase (OR=0.76).<br>– Carbohydrate: higher actual silhouette(OR=1.86); higher life increase (OR=1.56)	– Only women
**60**	Associations between weight misperception and weight-related attitudes and behaviours.	– NHANES (2003-2006)<br>– USA<br>– N=20.470 (BMI >25)<br>– Aged >20	– Measured height, weight and waist circumference.<br>**Body image:**<br>– Self-perception of overweight status<br>**Physical activity and energy intake:**<br>– Energy intake (two 24h diet recalls)	**Overweight prevalence** (50.5%)**:**<br>– Weight misperceivers: 23.0%.<br>– 80.6% wanted to lose weight; 47.4% attempted lose weight in the past year.<br>– Sedentary: 33.8%; insufficiently active: 25.2%; sufficient physically active: 41.0%.<br>**Weight management**<br>Overweight misperceivers compared to accurately perceivers:<br>– Want to lose weight: 71% M; 65% W.<br>– Not attempted to lose weight the past year: 60%M; 56%W.	– Includes young people

			– Physical activity<br>**Weight management:** desire to weigh less, trying to lose/maintain weight in past 12 months.	– Physical activity: 32.0% men less likely to be insufficiently active.	
**60**	Association between perceived overweight and weight control.<br><br>Discrepancies between perceived and measured weight.	– NHANES (2003-04 + 2005-06 + 2007-08)<br>– USA<br>– N=16.720<br>– Aged >18	– Measured height, weight and waist circumference.<br>**Body image:**<br>– Self-perception of overweight status<br>– Reporting medical diagnosis of overweight/obesity<br>**Weight management:** desire to weigh less, trying to lose/maintain weight in the past 12 months, weigh control strategies.	**Desire to lose weight:**<br>– Overall: 64.3% (55.1% M and 73.2% W).<br>– Age group: <55 y-o: 53.7% M and 74.9% W. >55 y-o: 59.0% M and 69.5% W<br>– Perception: NW: 48.2% W. OW: 95.2% M and 98.3% W.<br>– Health care professional diagnosis of excessive weight: 91.4% M and 93.9% W.<br>**Pursuit of weight control:**<br>– Overall: 48.4% (39.6% M and 57.1% W).<br>– Age group: <55 y-o: 39.2% M and 60.5% W. >55 y-o: 40.7% M and 50.1% W.<br>– Overweight perception: 59.6% M (OR 3.74); 71.6% W (OR 2.82).<br>– Health care professional diagnosis of excess weight: 65.0% M; 75.1% W.<br>**Trying to lose weight at the moment:**<br>– Overall: 37.0%. (27.3% M; 46.2% W).<br>– Methods: Any: 60.3% M; 73.5%W. Diet + Exercise: 32.3% M;.38.6% W.	– Includes young people
**60**	Body size perceptions in a RCT to prevent deterioration of glucose tolerance with diet and physical activity.	– Norway (Pakistani women living in Oslo)<br>– N= 198 women<br>– Aged: 25-62 (BMI> 25)	– Measured height and weight<br>**Body image:**<br>– Stunkard's Figure Rating Scale (actual + in a woman of 45 years: good health, wealthy, ideal for Norwegian, Pakistani and Pakistani living in Norway)<br>– Body Image Discrepancy score	– Actual perceived body size: 5.7<br>**Health and wealth** (significantly different from actual perceived)**:**<br>– Health: mean: 2.9. Youngers selected lower silhouettes.<br>– Wealth: mean: 3.3. Low educated selected extreme silhouettes.<br>**Body silhouettes preferences** (bigger through smaller silhouette)**:**<br>– Pakistani > Pakistani living in Norway > Norwegian.<br>**Discrepancy score:** (self-perceived – preferred by Pakistani living in Norway)<br>– Positive: 79%. (higher percentage in higher BMI groups)<br>– Negative among BMI 25.0-27.5: 20%	– Only Pakistani women living in Oslo
**60**	Ethnic differences in the relationship between weight perception and weight management behaviours.	– NHANES (1999-2006)<br>– USA<br>– N= 11.319<br>– Aged >20 (BMI > 25)	– Measured height and weight<br>**Body image:**<br>– Self-reported body image (obese, overweight, normal weight)<br>**Weight management:** desired weight, weight loss attempts, and individual's attempts to maintain weight the past 12 months	**General weight management:**<br>– Misperceivers:<br>o Tried to lose weight 29.1%. Tried not to gain weight 29.7%.<br>o Desired weight: less 33.3%; same 63.3%.<br>– Accurate perceivers:<br>o Tried to lose weight 61.1%. Tried not to gain weight 52.5%.<br>o Desired weight: less 97.3%; same 2.5%.<br>**Weight management: ethnicities** (non-Hispanic Blacks/Whites and Hispanics)<br>– Misperceivers<br>o Tried to lose weight: non-Hispanic Whites < non-Hispanic Blacks	– Includes young people<br>– Self-reported data about dieting

				o Desired weight less: non-Hispanic Whites < non-Hispanic Blacks<br>– Accurate perceivers:<br>o Desired weight less: non-Hispanic Whites < non-Hispanic Blacks	
**60**	Effect of eating on body image satisfaction.	– UK<br>– N=46 women in a commercial weight loss group.<br>– Mean age: 36,7 ±12,9	**Body image:**<br>– Body Shape Questionnaire (BSQ-16)<br>– Visual analogue Scales<br>– Diary for weight and body satisfaction, calorie intake and mood.<br>**Others:**<br>– Restraint Scale<br>– Appearance Schemas Inventory Revised	**Weight and shape dissatisfaction:**<br>– Correlated to: variability in perceived calorie content (r = - 0.30)<br>**Mean body image satisfaction correlated to:**<br>– BMI (r=-0.47)<br>– Weight and shape dissatisfaction (r=-0.40)<br>– Dietary restraint (r=-0.65)<br>– Variability in perceived calorie content (r=0.35).	– Includes young people<br>– Age range unspecified<br>– Low sample size
**60**	Relationship between ideal weight perception, weight satisfaction and health practices (weight cycling, physical activity, and diet)	– Aerobics Center Longitudinal Study (1997 + 2001)<br>– USA<br>– N= 19.347 (15221 men + 4126 women)<br>– Aged 20-87	– Anthropometric measurements (height, weight and skinfolds)<br>**Body image:**<br>– Weight history and ideal weight<br>– Weight satisfaction (y/n)<br>– Intention to lose weight (y/n)<br>**Physical activity and energy intake:**<br>– Dietary habits (fruit and vegetables servings) and eating behaviours<br>– Diet and physical condition satisfaction (y/n).<br>Intent to change physical condition (y/n) or diet (y/n)	**Body weight satisfaction:**<br>– BMI was associated with a lower odds of weight satisfaction (OR: 0.49 M; 0.47 W) and intentions to change weight (OR: 1.85 M; 2.10 W).<br>– Higher ideal weight was associated with a higher odds of body satisfaction (OR: 1.07 M; 1.09 W)and less intention to lose weight (OR: 0.95 M; 0.94 W)<br>**Physical activity and energy intake:**<br>– Weight satisfaction was associated with higher physical activity, higher fruits and vegetables intake,higher cardiorespiratory fitness and lowerintention to change diet.<br>– Intention to change diet: 93% men and 95% women dissatisfied with their weight.<br>– Lower fruit and vegetable consumption: OW men dissatisfied with their weight and women (regardless of the BMI) dissatisfied with their weight.<br>OW men dissatisfied with their weight consumed less fruits and vegetables.	– Unclear questionnaires<br>– Age unspecified (checked other study) + includes young people
**60**	Association of weight perception and participation (baseline) in an intervention promoting	– USA<br>– N=899 employees of hospitals<br>– Aged 18-65	– Measured height and weight<br>**Body image:**<br>– Current weight (7 item Likert-scale)<br>**Weight management:** currently trying to lose weight and strategies used.	**BMI prevalences:**<br>– Men: OB: 31.5%; OW: 47.1%; NW: 21.4%.<br>– Women: OB: 36.1%; OW: 28.1%; NW: 35.5%.<br>**Perceptions:**<br>– Men: OB: 24.3%; OW: 42.8%; NW: 23.8%; UW: 9.2%.<br>– Women: OB: 54.8%; OW: 38.4%; NW: 15.5%; UW: 5.4%.<br>**Weight management:**<br>– Currently trying to lose weight: 62.5%. (45% NW; 65% OW; 78% OB).	– Includes young people<br>– Employees of hospitals (people working in health care)

	weight control through lifestyle changes.			o Diet and or Physical activity: 43.7% (29.0% M; 47.6% W); bariatric surgery: 2%.<br>**Perceived weight status and weight loss attempts**<br>– Slightly OW perception: Men: OR=14.4; women: OR=8.9.<br>– Moderately OW perception: Men: OR=13.8; women: OR=8.2.<br>– Very OW perception: Men: OR=9.6; women: OR=5.0.	
**60**	Prevalence of meeting recommended levels of leisure-time physical activity and fruit and vegetable consumption.<br><br>Effects of weight status and perceptions.	– Australian National Health Survey (2004-05)<br>– Australia<br>– N=16 314 (7720 men, 8594 women)<br>– Aged > 20	– Self-reported height and weight<br>**Body image:**<br>– Self-reported weight perception (obese, overweight, normal weight)<br>**Physical activity and energy intake:**<br>– Daily fruit and vegetable intake<br>– International Physical Activity Questionnaire	**≥ 2 servings fruit / day:**<br>– OW: NW perception: 50.7%M; 61.9%W. OW perception: 46.1%M; 63.4%W<br>o OW itself (OR: 1.11) and OW perception in OW (OR: 1.14) increased the likelihood of having ≥ 2 servings fruit / day for women.<br>o OW perception in OW men (OR: 0.83) decreased the likelihood of having ≥ 2 servings fruit / day<br>– OB: NW perception:52.8 %M; 59.5%W. OW perception: 45.2%M; 62.4%W<br>**≥ 5 servings vegetables / day:**<br>– OW: NW perception: 14.4%M; 17.5%W. OW perception: 11.6%M; 18.5%W<br>– OB: NW perception: 14.8%M; 18.6%W. OW perception: 10.8%M; 18.2%W<br>**Physically active**<br>– Excess weight (except for OW men) and OW perception were related to less exercise.<br>**Physically active + adequate intake of fruits and vegetables in the past two weeks:**<br>– OW: NW perception: 3.6%M; 4.3%W. OW perception: 3.2%M; 4.0%W<br>OB: NW perception: 2.5%M; 5.0%W. OW perception: 2.1%M; 3.4%W	– Includes young people<br>– Self-reported height and weight<br>– Secondary objective<br>– Results may be attributable to global misperceptions related to health.(I.E.: overweighed perceiving themselves as normal weighed may believe they engage in healthy behaviour)
**60**	Body image, attitudes toward overweight people, and dietary behaviours.	– Taiwan<br>– N= 96 women (50 obese + 46 normal weight)<br>– Aged: 20-59	– Measured height and weight<br>**Body image:**<br>– Self-reported body image (obese, overweight, normal weight)<br>**Others:**<br>– Attitudes towards overweighed people.<br>– General questions about dietary practices (5 item Likert-scale).	**Perceived BMI:**<br>– NW group: OB: 11%; OW: 61%; NW: 28%.<br>– OB group: OB: 76%; OW: 22%; NW: 2%.<br>**Attitudes towards obesity** (%agreement)<br>– Employers should not hire overweight people. 56.5% NW. 76.0% OB.<br>– Overweight people are unattractive. 78.3% NW. 94.0% OB.<br>– All had a negative attitude towards obese people<br>**Dietary behaviours**	– Only women<br>– Includes young people<br>– Low sample size

				– OB group showed less control over portion size, fried foods and sugar-containing beverages	
**60**	Associations between weight expectations and anthropo-metric profile.<br><br>Psychological and eating factors of realistic weight expectations	– Canada<br>– N= 154 women<br>– Mean age 42,4 ± 5,6 (BMI 25-35)	– Measured height and weight.<br>**Body image**<br>– Goals and Relative Weights Questionnaire<br>– Body-Esteem Scale<br>**Weight management:** diet and weight history<br>**Others:**<br>– Three-Factor Eating Questionnaire	– Initial weight – happy weight: 18.6% (on average)<br>**Correlation between current BMI and:**<br>– Dream BMI: r = 0.49; ideal BMI: r = 0.67; Acceptable BMI: r = 0.71; Disappointed BMI: r = 0.80. (all significances below 0.001)<br>**Correlation between current BMI and ideal BMI:**<br>– Current BMI < 30.5 kg/m^2: r = 0.63; p < 0.0001<br>– Current BMI > 30.5 kg/m^2: r = 0.27; p = 0.02<br>**Diet and weight history:**<br>– Similar: age of first weight loss attempt, number of times dieting, highest and lowest weight during adult life,<br>**Satisfaction with their weight:**<br>– Realistic ideal BMI > unrealistic ideal BMI.	– Only women<br>– Includes young people
**60**	Impact of BMI, advertising and media on the nutrition transition and their impact on eating styles and body image.	– Jordan<br>– N= 800 women<br>– Aged 18-73	– Measured height and weight.<br>**Body image:**<br>– Stunkard’s Figure rating scales (actual, ideal, healthy, preferred by men)<br>**Others:**<br>– Motivation for Eating Scale.<br>– Sociocultural Attitudes towards Appearance Scale.<br>– Body Esteem Scale.	– Mean BMI: 26.1 kg/m2. Mean silhouette: 5.<br>**Average weight loss:**<br>– Desire to lose 1 silhouette and become silhouette 4 (healthiest silhouette).<br>– 5.4% wanted to be larger than 5; 8.5% wanted to be thinner than 3.<br>**Desire to lose weight:**<br>– Media pressure: increases 2.908 times the likelihood of wanting to lose weight.<br>– Compared to NW:<br>o OW increases 11.14 times the likelihood of wanting to lose weight.<br>o OB increases 18.05 times the likelihood of wanting to lose weight.	– Only women<br>– Includes young people<br>– Main objective: pathological disorders related to body image (not weight restraint)
**60**	Perception of weight, attempts to lose weight.	– BEACH program<br>– Australia<br>– N= 1973 (1144 women + 829 men)<br>– Aged > 18	– Self-reported height and weight<br>**Body image:**<br>– Weight status self-perception<br>**Weight management:**<br>– Attempts to lose weight in the past 12 months<br>– Weight loss methods used in the past 3 years	**% overweight perceivers:** 87.5% OB; 59.6% OW; 15.5% NW; 3.6% UW.<br>**Weight management:** 37.0 % Attempted to lose weight in the past 12 months<br>**Weight management strategies** (% use)**:**<br>– Diet or exercise: 56.6% OB; 40.0% OW; 20.0% NW.<br>– General practitioner advice: 26.2%OB; 11.7% OW; 2.4% NW.<br>**Successful strategy:**<br>– Diet-exercise: 40.6% OB; 50.2% OW.<br>– No successful method: 34.7% OB; 27.5% OW.	– Includes young people<br>– Objective not clearly defined
**60**	Influence of weight loss	– QUOVADIS study	– Measured weight andheight.<br>**Body image:**	**Weight goals predictors:**<br>– Age; BMI. Body image was not a predictor	– Includes young people

	expectations on attrition.	– Italy<br>– N= 1785 obese (392 men + 1393 women)<br>– Aged 25-65	– Body Uneasiness Test<br>– Expected 1-year loss (after treatment)<br>– Maximum acceptable and ideal weight after treatment	**Attrition predictors:**<br>– Age (12 months. β=-0.019)<br>– Expected 1-year loss (6 months: HR=1.19; 12 months: HR=1.12). Attrition risk increase 12% per 1 kg/m² desired loss<br>– Body image (6 and 12 months: HR=1.42)<br>**BMI loss predictors:**<br>Baseline BMI (β=0.28); age (β=-0.16); age at first dieting (β=-0.10). Body image was not a predictor	– Main objective: drop-outs, body image was secondary<br>– BMI at drop-out not systematically recorded.<br>– No information about longitudinal changes in weight goals<br>– Dietary treatment not homogenous.
**60**	Weight perception and management practices.	– USA<br>– N= 198 obese Latinos (Community: 90 women, 70 men; Labour camp: 38 men)<br>– Aged 18-64	**Body image:**<br>– Weight status self-perception<br>**Weight management:**<br>– Currently trying to lose weight and strategies used.<br>– Advice of health care provider.<br>**Others:**<br>– Acculturation to the US	**Perceived overweight:**<br>– Community: 68.6% M; 83.3% W; Labour camp: 73.7% M.<br>Among those consulting management strategies with a health care provider:<br>– Community: 80.5% M; 87.5%W;; Labour camp: 85.7% M.<br>**Weight management** (% currently trying to lose weight)**:**<br>– Community: 55.7% M; 72.2% W; Labour camp: 26.3% M.<br>Among overweight perceivers (% currently trying to lose weight):<br>– Community: 67% M; 75% W; Labour camp: 36%M.<br>Among those consulting management strategies with a health care provider:<br>– Community: 70.7% M; 73.6% W; Labour camp: 42.9% M.<br>**Weight management strategies** (% use)**:**<br>– Dieting: Community: 84.6% M; 93.8% W; Labour camp: 80.0% M.<br>– Exercising: Community: 61.5% M; 52.3% W; Labour camp: 50.0% M.<br>– Health care provider advice: Community: 59% M; 80% W; Labour camp: 37% M.	– Includes young people<br>– Self-reported data<br>– Secondary objective<br>– Telephone survey
**60**	To assess self-perception of body weight, attitudes toward	– Spain<br>– N= 1200<br>– Aged: 20-65	– Anthropometric measurements<br>**Body image:**<br>– Self-estimation of body weight (comparison of the estimated with the measured BMI).	**BMI prevalences:**<br>– OW: 27.8% (38.4% M; 25.1% W).(men vs women: p<0.01)<br>– OB: 13.1%; (10.4% M; 13.7% W).(men vs women: p<0.01)<br>**Perceived BMI:**	– Includes young people

	weight-control behaviours, and associated factors.		**Diet**<br>– Two 24-hour dietary recall<br>**Weight management:** Worries about effects of their excess weight on health, history of dieting, or how they considered their physical status.	– UW or NW: Underestimation: 2.7% M; 2.9% W; Overestimation: 9.1% M; 3.9% W.<br>– OW or OB: Underestimation: 15.3% M; 23.0% W; Overestimation: 0.0% M; 1.1% W.<br>**Overweight self-consideration** (Do you consider yourself overweight or obese?)**:**<br>– UW or NW: 1.8% M; 16.4% W.<br>– OW or OB: 52.0% M; 84.0% W.<br>**Weight management** (ever dieted)**:**<br>– UW or NW: 20.0% M; 48.0% W.<br>– OW or OB: 46.0% M; 84.7% W.	
**60**	Weight loss expectations in obese patients seeking treatment.	– QUOVADIS<br>– Italy<br>– N= 1891 obese patients seeking treatment (418 men + 1473 women)<br>– Aged 25-65 (BMI > 30)	– Anthropometric measurements<br>**Body image:**<br>– The Body Uneasiness Test<br>– Expected 1-year loss (after treatment)<br>– Maximum acceptable weight after treatment<br>– Ideal weight (realistic or not)<br>– **Weight management:** Reason for seeking treatment	– Mean BMI: 38.2± 6.5 kg/m² (38.0±6.7kg/m² M; 38.2± 6.4 kg/m² W)<br>**BMI loss expectations** (mean kg/m²; p<0.001 between sexes)**:**<br>– Maximum acceptable BMI: 29.3± 4.4kg/m² (30.2± 4.1kg/m² M; 29.0± 4.4kg/m² W)<br>– Dream BMI: 26.0± 3.4kg/m² (27.3± 2.7kg/m² M; 25.7± 3.5kg/m²W).<br>– Expected 1-year BMI loss: 10.2± 3.8kg/m² (8.8± 3.3kg/m² M; 10.6± 3.9kg/m² W).<br>– Expected 1-year loss: related to maximum previous lost (r_s=0.30; p<0.0001)<br>– BMI: strongest predictor of dream and maximum acceptable BMI.<br>**Weight loss motivation** (primary)**:**<br>– 15.2% Appearance (7.4% M; 17.4% W).<br>– 51.5% Present Health (56.0% M; 50.2% W).<br>– 33.4% Future Health (36.6% M; 32.5% W).<br>– In women: primary appearance motivation: lower current, dream and maximum acceptable BMI, younger, first attempted weight loss at a younger age (significant).	– Includes young people<br>– Men/women rate
**60**	Potential correlates of obesity (dietary intake, body image, and physical activity).	– Caretakers of children in the Hip-Hop to Health Jr. (5-year RCT)<br>– USA<br>– N= 305 women (271 black + 234 Hispanics)<br>– Aged 18 - 67	– Measured weight and height<br>**Body image:**<br>– Figure rating scales (actual, ideal).<br>– Question: Importance of body weight or shape on self-concept.<br>**Weight management:** Weight loss attempts<br>**Physical activity and diet related:**<br>– 24-hour dietary recall<br>– Regular physical activity	**BMI prevalences:** UW: 0.6%; NW: 22.2%; OW: 31.2%; OB: 45.9%.<br>– Mean BMI: Black: 31.4±8.4kg/m²; Hispanics: 29.6±5.9kg/m². (p<0.01)<br>**Body image** (figure rating scales)(p<0.01 between ethnicities)**:**<br>– Current body image: Black: 4.62± 0.07; Hispanics: 4.95± 0.08.<br>– Ideal Body image: Black: 3.54± 0.07; Hispanics: 3.14± 0.07.<br>– Body image discrepancy: Black: 1.09± 0.09; Hispanics: 1.80± 0.09.<br>**Weight management:**<br>– No regular physical activity: 55.6%<br>– Attending weight loss program: 98.4%<br>– Weight and shape were not important: 68.9%	– Only women<br>– Includes young people<br>– Only one 24-h recall
**60**	Levels of dietary	– Parents of participants	– Measured weight and height<br>**Body image:**	**Mean self-reported data:**<br>– BMI. Men: 26.7± 3.69 kg/m²; Women: 26.4± 4.81 kg/m².	– Age unspecified.

	restraint are associated with mis-reporting measures of adiposity.	in Barry Caerphilly Growth Study<br>– UK<br>– N= 227 (146 women and 81 men)<br>– Age unspecified (children aged 25-26y: middle-aged)	– Self-reported anthropometric measurements<br>**Diet:**<br>– 10 restraint questions from the Dutch Eating Behaviour Questionnaire	– BMI was associated with the mis-reporting of waist circumference for both genders.<br>**Mean difference** (self-reported – measured)**:**<br>– Weight. Men: -1.1 kg; Women: -2.3 kg. ($p<0.0001$)<br>– Height. Men: 1.9 cm; Women: 0.9 cm. ($p<0.0001$)<br>– BMI. Men: -0.9 kg/m² (r=0.28); Women: -1.2 kg/m² (r=0.38). ($p<0.0001$)<br>– Waist: Men: -4.3 cm; Women: -5.2 cm.($p<0.0001$)<br>**Dietary restraint score**<br>– Women: +1 Dietary restraint score = +0.36 kg/m² self-reported – measured BMI	– Self-reported weight and height (*validated)
**60**	Characteristics of misreporting of energy intake during 24-hour dietary recalls.	– USA<br>– N=98<br>– Aged 25-73	– Anthropometric measurements<br>**Body image:**<br>– Desired and ideal weight<br>– Overweight self-perception<br>– Weight loss attempts in the last year<br>**Energy intake:**<br>– Two 24h diet recall<br>– Energy requirements (doubly labeled water or intake balance)<br>– Energy intake (measured energy expenditure)	– Energy intake under-reporters: 61% M, 85%W.<br>**Weight perception:**<br>– -304 kcal underreported for considering oneself overweight or desire to weigh less.<br>– -15.6 kcal underreported per kg above ideal weight<br>**Weight management** (tried to lose weight in the past year)**:**<br>– -339 kcal underreported for desire to weigh less.<br>– -398 kcal underreported for having attempted weight loss<br>– -17 kcal underreported per kg gained in the past 10 years.	– Low sample size<br>– Includes young people<br>– Energy intakewas measured as energy expenditure (they can be trying to lose weight or getting fatter)
**60**	Weight perceptions and weight control are associated with body weight.	– Omnibus Survey of the Office of National Statistics (1999)<br>– UK<br>– N= 1799 (896 women + 903 men)<br>– Age >16	– Self-reported anthropometric measurements<br>**Body image:**<br>– Report actual perceived and ideal body weight<br>**Weight management:** Current and in the past 3 years weight control. Methods used.	**Weight perception** (Gender differences)**:**<br>– UW perception:<br>Men: 53.9%UW; 15.5%NW; 0.3%OW. Women: 54.1%UW; 7.0%NW; 0.4%OW; 1.8%OB.<br>– OW perception:<br>Men: 14.2% NW; 67.6% OW; 97.2% OB. Women: 28.3% NW; 89.2% OW; 94.5% OB.<br>– Association with BMI: Men: r=0.70; Women: r=0.76.<br>**Ideal weight** (In the range of normal weight. Higher among men)**:**<br>– Ideal < actual: men: OW, OB; women: OW, OB, NW (*NW: low desire weight change).<br>– Ideal = actual: men: NW.<br>– Ideal > actual: men: UW; women: UW.<br>**Weight control:**	– Self-reported weight and height<br>– Includes young people<br>– Telephone survey<br>– Term “about right” is not so clear.

				– Correlations to trying to lose weight: BMI: men: r=0.42; women: r=0.48<br>Perceived BMI: men: r=0.52; women: r=0.56<br>– Weight loss attempt in the past 3 years:<br>o Men: 16.3% NW; 44.2% OW; 66.7% OB<br>o Women: 8.1% UW; 44.4% NW; 71.3% OW; 80.0% OB<br>**Methods**: Diet: 26.2% M; 51.5% W. Advice from professional: 3.0% M; 4.4% W	
**60**	Erroneous expectations about the likely outcomes of weight loss diets.	– N=62 women: 30 in androgynous group (waist-to-hip ratio>0.82) + 32 in pear group (waist-to-hip ratio < 0.76)<br>– Mean age: 33 ± 9	– Measured hip and waist.<br>**Body image:**<br>– 5 computer generated pairs of images increasing BMI, differing between pairs only in hips and thighs.<br>– Diet outcome: 5 times each image (randomly)<br>– Current body: all at the same time	– Both groups were accurate selecting current body shape.<br>– Both groups (androgynous and pear-shaped) chose the pear-shaped image as diet outcome	– Only women<br>– Real age unspecified + Includes young people. (95% included in the range of 15-51y)
**60**	Dieting history, measured and perceived weight.	– MORGEN (1989)<br>– Netherlands<br>– N= 4601 (2155 men + 2446 women)<br>– Aged 20-59	– Measured weight and height<br>**Body image:**<br>– Question: 'Describe your weight?'<br>**Weight management:** History of loss of 5Kg by dieting, current weight control status.	– Mean BMI: 25.4± 3.6 kg/m² M; 24.5± 4.2 kg/m² W<br>**Weight perception:**<br>– Men: 5.9% UW perception; 54.8% NW perception; 39.3% OW perception.<br>– Women: 3.1% UW perception; 51.8% NW perception; 45.1% OW perception.<br>**Weight control:**<br>– Last year weight loss attempts:<br>o Men: 79.2% never; 13.4% once; 7.5% twice or more.<br>o Women: 62.8% never; 20.2% once; 17.1% twice or more.<br>– 5kg loss<br>o Men: 74.8% never; 18.1% once or twice; 7.2% more than twice.<br>o Women: 59.2% never; 26.1% once or twice; 14.6% more than twice.	– Cross sectional design<br>– Includes young people
**60**	Attitudes about weight, appearance and food and nutrient intakes.	– USA<br>– N=221 women<br>– Aged 50-75	**Body image:**<br>– Weight concerns (Haynes and Ross)<br>**Energy intake:**<br>– National cancer institute 60-item Food frequency questionnaire.	– Food intake provided the RDA for all nutrients except energy, calcium and iron.<br>– BMI was positively associated to weight concerns (r=0.20) and inversely associated to appearance concerns (r=-0.18) and the importance of nutrition (r=-0.23).<br>– Age was inversely related to weight concerns (r=-0.22)<br>**Food intake and body image:**<br>– Weight concerns were associated to higher fish and chicken consumption (r=0.23) and the association to a higher fibre intake was suggested.	– 1991-92<br>– Only women<br>– Telephone survey<br>– Does not consider measured BMI

				– Appearance concerns were associated to lower % of energy as fat (r=-0.13).	
**60**	Weight status perception, accuracy of self-assessment of weight status and weight control practices relative to the degree of adiposity.	– MORGEN project (1995)<br>– Netherlands<br>– N=4601 dieters (2155 men and 2446 women)<br>– Aged 26-65	– Anthropometric measurements<br>**Body image:**<br>– Perception of weight status<br>– Ideal body weight<br>**Weight management:** History of weight loss and dieting, current weight control status, methods and reasons for attempted weight loss<br>**Physical activity and diet related:**<br>– Question: "do you engage in sport?"<br>– Semi-quantitative food frequency questionnaire	**Weight perception:**<br>– NW: *UW perception*: 10.7% M; 3.3%W. *OW perception*: 10.2% M; 23.4% W.<br>– Healthy OW: *UW perception*: 0.2% M; 0.2%W. *OW perception*: 54.3% M; 79.3% W.<br>– Not healthy OW: *OW perception*: 62.6% M; 69.7% W.<br>– OB: *UW perception*: 0.5% M. *OW perception*: 91.4% M; 97.2% W.<br>**Women over 40 y-o were less likely to self-perceive OW or OB than younger (OR=0.7)<br>**Current weight control:**<br>– NW: *Lose*: 81% M; 23.0%W. *Maintain*: 21.5% M; 33.2% W. *Gain*: 7.7% M; 2.4% W.<br>– Healthy OW: *Lose*: 33.7% M; 56.0% W. *Maintain*: 23.0% M; 23.0% W. *Gain*: 0.2% M.<br>– Not healthy OW: *Lose*: 35.1%M; 53.8%W. *Maintain*: 19.5%M; 18.9%W. *Gain*: 0.3%M.<br>– OB: *Lose*: 59.4% M; 63.0% W. *Maintain*: 9.6% M; 10.8% W. *Gain*: 0.5% M.<br>**Methods**: Main: diet. Rare: physical activity. Professional advice: 5% M; 7% W.<br>**Reasons**: OW or OB individuals: health. NW individuals: appearance.	– 1998<br>– Includes young people.<br>– Methods (questionnaires) unspecified.
**60**	Realistic shape perceptions, current and ideal or attractive shape and weight perceptions.	– USA<br>– N=20.000 dieters (after 3 years of diet)<br>– Mean age: 45 ±15.	– Self-reported weight and height<br>**Body image:**<br>– Body silhouettes (actual, ideal, smallest realistic, male and female attractive, attractive to the other gender)<br>– Ideal and reasonable weight	**Weight perception** (significant differences between men and women)**:**<br>– Ideal shape < realistic shape < current shape (for all).<br>– Ideal and realistic: lower for women. Dissatisfaction: (Real – ideal): bigger for women.<br>– Reasonable weight loss: higher than ideal weight (8.7% M < 14.7% W).<br>– Men: accurate male attractive figure. Women: inaccurate in female attractive figure<br>– No race differences were found.	– 1998<br>– Includes young people.<br>– Self-reported weight and height.<br>– All ethnics (good).
**60**	Weight, body image, and weight control practices	– Navajo Health and Nutrition Survey<br>– Navajo Indians (USA)<br>– N=786<br>– Aged >20	– Anthropometric measurements (weight, height and skinfolds)<br>**Body image:**<br>– Asked to classify themselves as UW, NW or OW.<br>– Silhouettes adapted to Navajo Indians (ideal).<br>**Weight management:** Weight control practices	**BMI prevalences:**<br>– UW: 20-39 y-o: 7% M; 3% W. 40-59 y-o: 5% M; 1% W. > 60 y-o: 14% M; 2% W.<br>– OW: 20-39 y-o: 37% M; 62% W. 40-59 y-o: 50% M; 69% W. > 60 y-o: 9% M; 61% W.<br>**Weight perception:**<br>– 19% underestimation; 17% overestimation (17% M; 21%W)<br>– Overestimation across age group: 20-39 y-o: 21%; 40-59 y-o: 13%; > 60 y-o: 11%.<br>– Ideal weight of OW: 20-59 y-o: 14% M; 7% W. > 60 y-o: 31% M; 17% W.<br>**Current weight control:**<br>– 43%. 35% M (26% NW; 57% OW); 48% W (16% UW; 35% NW; 57% OW).	– 1997<br>– Includes young people.<br>– Only Navajo Indians

				– Age: 20-39 y-o: 43% M; 59% W. 40-59 y-o: 38% M; 43% W. > 60 y-o: 10% M; 29% W.<br>**Methods**: Diet: 82%; pills: 3%, other products: 3%; vomiting after eating: 4%	
**60**	Body image perceptions and attempts to alter weight.	– USA<br>– N=370 diabetic African Americans (224 men + 146 women)<br>– Mean age: 50 ±13	**Body image:**<br>– Body silhouettes adapted to African Americans (actual, ideal, dietitian's objective, ideal (on you) for the most important adult in your life)<br>**Weight management:** Attempts to change weight and amount archived	**Weight perception:**<br>– Correlation perceived size and BMI: Men r=0.77; Women: r=0.76.<br>– Satisfaction: NW: 58% M; 56% W. OW: 21% M; 13%M. In OW women satisfaction correlated negatively to age (r=-0.24)<br>– Correlation perceived size and satisfaction: OW Men r=-0.33; OW Women: r=-0.34.<br>– Desired own size: OW > NW for both men and women.<br>– Desired own size – current: OW > NW; trying to change weight>not trying; bigger for women.<br>– Desired by dietitian > current for women and OW men. Bigger for women.<br>– Desired own size > dietitian desired for men and OW women.<br>**Current weight control:**<br>– NW: 54% M; 43% W. OW: 83% M; 88%W.<br>– Perceived size correlated to weight control in OW (Men r=0.3; Women r=0.2)<br>– Body size satisfaction was negatively related to weight control.	– 1997<br>– Real age unspecified + Includes young people
**60**	Accuracy of self-reported weight.	– Baseline from a worksite health promotion (1988-91)<br>– USA<br>– N=4432 (2041 men + 2391 women)<br>– Mean age: 38 ±10	– Measured weight.<br>**Body image:**<br>– Self-reported weight<br>– Weight concern defined as questions about dieting.	**Weight underreporting:** 1.3% M; 1.7%W. (Weight was the strongest predictor)<br>– High weight and low height were related to weight underreporting.<br>**Weight control related to weight underreporting:**<br>– Following a weight loss programme was related to weight underreporting.<br>– History of weight loss was related to weight underreporting.<br>– History of weight related diseases was related to weight underreporting in men.	– 1996<br>– Real age unspecified + Includes young people<br>– Only employed people
**60**	Attitudes toward eating and body size perceptions	– Charleston Heart Study (1991)<br>– USA<br>– N= 517 women<br>– Aged 66-105	**Body image:**<br>– Stunkard scale (actual perceived + ideal body size)<br>– Questionnaire developed to examine eating attitudes and body size perceptions (actual perceived + ideal body size)	**Weight perception:**<br>– Perceived current silhouette: white > black. OW > NW.<br>– Discrepancy score (ideal-perceived weight): Black: 0.3; White: 1.4; NW: 0.3; OW: 1.6.<br>– Ideal BMI – perceived BMI: Black: 1.0; White: 2.8; NW: 0.2; OW: 4.7.<br>– Ideal BMI: Black NW: 23.8 kg/m^2; OW: 28.7 kg/m^2.<br>**Weight management** (differences in weight categories):	– 1991<br>– Only women<br>– Methods not clarified in the article.

				– Current weight satisfaction: Black: 79% NW; 58% OW. White: 70% NW; 26% OW.<br>– OW self-perception: Black: 7% NW; 70% OW. White: 41% NW; 94% OW.<br>– OW society perception: Black: 0% NW; 46% OW. White: 19% NW; 62% OW.<br>– Feeling attractive: White: 67% NW; 37% OW. No differences in black women (71.5%).<br>– Ever tried to lose weight: White: 49% NW; 86% OW. No differences in black (52.5%).<br>– Ever tried to gain weight: Black: 16% NW; 5% OW. White: 22% NW; 10% OW.	
**60**	Examine body size perceptions, dieting and cognitive control of intake ~~Impact of race, weight status and education~~	– Charleston Heart Study (1991)<br>– USA<br>– N=334 men<br>– Aged 55-98	– Measured weight and height<br>**Body image:**<br>– Stunkard scale (actual perceived + ideal body size)<br>– Questionnaire developed to examine eating attitudes and body size perceptions (actual perceived + ideal body size)	**Weight perception:**<br>– Perceived current silhouette: white > black. OW > NW.<br>– Discrepancy score (ideal-perceived weight): Black: 0.4; White: 0.9; NW: 0.2; OW: 1.5.<br>– Ideal BMI – perceived BMI: Black: 0.7; White: 1.3 NW: -0.2; OW: 2.9.<br>– Ideal BMI: NW: 24.2 kg/m^2; OW: 28.5 kg/m^2. Black: 26.1 kg/m^2; White: 25.6 kg/m^2.<br>**Weight management** (differences in weight categories)**:**<br>– Current weight satisfaction: Black: 83% NW; 50% OW. White: 69% NW; 42% OW.<br>– OW self-perception: Black: 29% NW; 52% OW. No differences in white men (59%).<br>– OW society perception: Black: 5% NW; 60% OW. White: 26% NW; 63% OW.<br>– 78% feel attractive.<br>– Ever tried to lose weight: 37% Black (42% NW; 33% OW). 64% White (49% NW; 82% OW). Differences in weight categories and ethnic groups. (Black: affected by education)<br>– 15% ever tried to gain weight (Black: affected by education).	– 1991<br>– Only men
**60**	Diet enrolees' initial body image compared to controls	– "New Direction" (very low calorie) by Ross Laboratories + Cash (1991)<br>– USA<br>– N=360 (102 dieters +258 control)	– Measured weight and height<br>**Body image:**<br>– 69-item Multidimensional Body-Self Relations Questionnaire.	**Weight satisfaction:**<br>– Dieters reported less satisfaction with their appearance and body.<br>**Health:**<br>– Dieters felt less healthy than controls (objective health was similar for both cohorts)<br>– Dieters felt fitter than controls.<br>– Dieters were more conscious about their appearance and fitness.	– 1993<br>– Real age unspecified + Includes young people<br>– Mainly women.<br>– Some morbid obese dieters

		– Age: 41,4 ±10,3			– Sponsored diet program
**60**	Knowledge and practices regarding weight loss, trends between 1985-90.	– NHIS<br>– USA<br>– N=55545 (24171 in 1985 +31374 in 1990)<br>– Aged >25	**Body image:**<br>– Self-perceived weight status on those trying to lose weight.<br>**Weight management:** Methods to lose weight.	**Weight perception:**<br>– OW: 36.7% M; 52.0% W. 25-44 y-o: 43.8%; 45-64 y-o: 52.4%; > 65 y-o: 35.7%.<br>– UW: 6.2% M; 4.5% W. 25-44 y-o: 5.1%; 45-64 y-o: 3.7%; > 65 y-o: 8.6%.<br>– Ethnicities: black persons were the less likely to consider themselves as OW.<br>**Weight management** (trying to lose weight)**:**<br>– 23% M; 40%W. 25-44 y-o: 35.5%; 45-64 y-o: 34.7%; > 65 y-o: 19.7%.<br>– Weight perception: OW: 58.6%; NW: ~12% (1985 > 1990). UW: ~ 2% (1985: < 1990).<br>**Weight management strategies** (% use)**:**<br>– Methods (from best to worst): Eat few calories > not eating before going to bed > physical activity > others. If combining: few calories + physical activity.<br>– Eating less: 76.4% M; 83%W. 25-44 y-o: 78.9%; > 45 y-o: 82.5%.<br>– Increasing activity: 60% M; 57.5% W. 25-44 y-o: 64.5%; 45-64 y-o: 53%; > 65 y-o: 40.5%.	– 1993<br>– Includes young people<br>– Self-reported height and weight<br>– Telephone survey<br>– No comparison Body image with anthropometry (only ask to classify them in weight group, nor to report the actual weight/height)
**60**	Relevant factors to design and implement weight control programs.	– USA<br>– N=500 black women<br>– Aged 25-64.	– Reported weight and height<br>**Body image:**<br>– Self-developed exploratory questions (identifying new perspectives on weight control).<br>**Weight management:** Weight related attitudes and behaviours	**Weight perception:**<br>– Weight satisfaction: OB: 3%; OW: 5%; NW: 19%; UW: 47%.<br>– Feeling attractive: OB: 39%; OW: 44%; NW: 74%; UW: 85%. Predictor: younger age.<br>**Weight management strategies:**<br>– Currently trying: OB: 37%; OW: 31%; NW: 25%; UW: 14%.<br>– Ever tried: OB: 90%; OW: 88%; NW: 78%; UW: 41%. Motive: health and appearance.<br>– Strategies: Diet 39% (in UW: 28%); Exercise: 12%; Diet + exercise: 45%.<br>**Weight maintaining:**<br>– Methods used (% maintainers): exercise: 79%; diet: 60%; diet + exercise: 55%.<br>– 64% tried alone, 38% in a weight loss group.<br>– Weight regainers were 2.7 times as likely to report eating between meals.	– 1993<br>– Includes young people<br>– Only women<br>– Self-reported height and weight<br>– Does not specify the body image assessing technique

**60**	Relationship between height and body image parameters related to dieting, body weight and shape.	– Canada<br>– N=174<br>– Mean age: 40 ±15,7	– Reported height and weight<br>**Body image:**<br>– Eating Disorder Inventory (2 subscales: drive for thinness and body dissatisfaction)	– Drive for thinness and body dissatisfaction scores were in the normal range.<br>– Women had more concerns about both, body shape and weight management.<br>**Body shape concerns:**<br>– Positively correlated to BMI: Men (r=0.39). Women (r=0.51)<br>– Negatively correlated to high in men (r=-0.25) (independent of body weight)<br>**Weight management:**<br>– Positively correlated to BMI in women (r=0.27)<br>– Negatively correlated to high in men (r=-0.25) (independent of body weight)	– 1993<br>– Real age??<br>– No diet considered<br>– Self-reported height and weight<br>– Related somehow to eating disorders (questionnaire)
**60**	Risk behaviours of the overweight compared with average weight. Association between body image, weight status, and dieting.	– BRF 1981-1983<br>– USA<br>– N=19405<br>– Aged 18-79	– Reported height and weight.<br>**Body image:**<br>– Considered themselves overweight, underweight, or average<br>**Weight management:** Currently on a diet (maintain or lose weight)	**OW prevalence:**<br>– 23% OW: 23.9% M; 22.2% W. Whites: 21.8% Black and Hispanic: 31.2%<br>– Increases with age: reaching its peak at 60 y-o and descending after that age.<br>**Weight perception:**<br>– Accurate weight perception: OW women > OW men. White > black.<br>**Weight management:**<br>– Dieting is related to weight status (28% OW Men; 48% OW Women).<br>– More females than males are dieting within each weight group.<br>– OW perception is related to dieting.<br>– The proportion of overweight on a diet increases with perception of weight status.	– 1986<br>– Includes young people<br>– Self-reported height and weight<br>– Telephone survey<br>– No BMI → % weight for height

Abbreviations: BMI: Body Mass Index. UW: Underweight, NW: Normal weight, OW: Overweight, OB: Obese. M: Man, W: Women. y-o: years – old.

Table S2. Intervention studies.

Ref.	Objective	Sample	Intervention	Evaluation methods	Results	Limitations
**67**	Effect of a personalised future self-image on weight change over a 6-month period.	– Australia<br>– N= 121<br>– Aged: 18-79<br>– BMI: >25 (*measured)<br>– 24 weeks	– Time of watching future self-image (image of the volunteer in the future, if the same lifestyle continues) + late new image (randomized):<br>1. At baseline + new image<br>2. At baseline + No new image<br>3. 8 weeks delayed + new image<br>4. 8 weeks delayed + No new image<br>– All received 15 min of lifestyle advice (reduce intake and increase physical activity). Participants chose weight management method. Follow up measurements every 4 weeks.	– Measured weight and height.<br>**Body image:**<br>– Computer designed future self-image	– 69.4% of participants finished the study<br>**Weight loss:**<br>– Very significant weight change overall.<br>– week 16: delayed-image: −0.50%; early-image: −0.30%<br>– week 24: delayed-image: −0.50%; early-image: −0.27%<br>– Greater weight loss for the delayed-image group.<br>– Late (second) image did not influence weight.	– Includes young people.<br>– High dropout rate<br>– Measure of the effect of a future image on lifestyle and weight (other way around)<br>– Very light lifestyle intervention<br>– No control group
**61**	Best treatment (weight, physical activity, body satisfaction, and healthy eating).<br><br>Association between physical activity	– USA<br>– N=107 physically inactive women. (53+54)<br>– Aged 30-65<br>– BMI: 30-40 (*measured)<br>– 24 months	– Treatment 24 weeks<br>– All encouraged to increase their physical activity (150 min/week)<br><br>Randomly assigned to:<br>o The Coach Approach exercise-support protocol (group sessions emphasizing physical activity-derived self-regulation) (n=53)<br>o LEARN (low fat) Program for Weight Management (written manual) and phone support (n=54)	Follow up: 3, 6, 12, 24 months<br>– Measured weight and height.<br>**Body image:**<br>– Body Areas Satisfaction Scale of the Multidimensional Body-Self Relations Questionnaire<br>**Physical activity**<br>– Godin—Shephard Leisure-Time Physical Activity Questionnaire	**Body satisfaction predicted body weight change:**<br>– 3 month satisfaction predicted 6 month weight (β=-0.35)<br>– 6 month satisfaction predicted 12 month weight (β=-0.51)<br>– 12 month satisfaction predicted 24 month weight (β=-0.41)<br>**Body weight change predicted body satisfaction:**<br>– 3 month weight predicted 6 month satisfaction (β=-0.34)<br>– 6 month weight predicted 12 month satisfaction (β=-0.42)<br>– 12 month weight predicted 24 month satisfaction (β=-0.47)<br>**Physical activity**<br>– Changes in physical activity related to body satisfaction, even after controlling for weight change and treatment.	– Only women<br>– Includes young people

	and body satisfaction .					
**63**	Develop and test the effectiveness of the programme.	– USA<br>– N= 90 (38 standard care + 52 continuing care)<br>– Aged 20-72<br>– BMI: 26-65 (*measured)<br>– 24 months	– 2 groups: 20 sessions (6 months) focused on behaviours modification, healthy eating and physical activity. (Diabetes Prevention Program)<br>– Participants in continuing care were taught self-support strategies and instructed to continue meeting weekly on their own throughout the 18 months following treatment, while the control group was neither advised nor dissuaded to continue meeting.	Follow up: baseline, 6, 12 and 24 months<br>– Measured weight and height.<br>**Body image:**<br>– Body Shape Questionnaire (14-item version)<br>**Diet and physical activity**<br>– Eating Habit Questionnaire<br>– International Physical Activity Questionnaire short-form	**Weight**:<br>– 24 months: 25% of initial weight regained<br>– Differences between baseline and each follow up, but not among follow ups.<br>**Diet**<br>– Significant differences for time in all subscales of the Eating Habit Questionnaire: Meat (f=9.26), Fat (f=6.93), Vegetables (f=4.35), Modifications (f=15.17), and Total (f=15.98).<br>**Physicalactivity**<br>– Walking: baseline and post-treatment differences<br>– Moderate activity: baseline and 6 months differences<br>**Body image**<br>– Significant effect for time. Body image improves in continuing care group. (f=8.30)	– Includes young people<br>– Includes morbid obsesses<br>– Secondary objective.<br>– There was no difference between groups in meetings after the treatment, which was the main difference between groups.
**66**	Changes in obesity-related attitudes.	– USA<br>– N= 123 women<br>– Mean age: 44,2 ±10,0<br>– BMI: 30-46<br>– 40 weeks	– Weekly group sessions<br>– All were encouraged to increase their physical activity to 180 min/week<br><br>Diet therapy (randomized):<br>o Diet therapy either by meal replacement (Optifast 800) or by balanced hypocaloric self-selected diet (1200 to 1500 kcal/d; 30% fat)<br>o Non-dieting program (Eating healthy + forgetting about weight or calories)	Follow up: baseline, 20, 40 weeks.<br>– Measured weight and height.<br>**Body image:**<br>– Body Shape Questionnaire<br>**Others:**<br>– Sociocultural Attitudes Toward Appearance Questionnaire<br>– Rosenberg Self-esteem Scale	**Weight**(significant differences among groups):<br>– Dieting group: week 20: 6.0%: week 40: 8.4%.<br>– Non-dieting group: week 20: 2.5%: week 40: 3.2%.<br>**Body image improvement**(Improvement through time)**:**<br>– Dieting group: week 20: 18.4%: week 40: 22.0%.<br>– Non-dieting group: week 20: 15.9%: week 40: 26.2%.<br>– *No differences between groups.<br>**Body image correlated with:**<br>– Self-esteem (week 20: r=0.23; week 40: r=0.36)<br>– Less internalization of society's appearance ideals (week 40: r=0.35)	– Real age unspecified + includes young people<br>– Mainly women.<br>– Some morbid obese dieters<br>– Main objective: to study attitudes towards obesity, body image is secondary.
**44**	Body image	– USA	– Diet: medically supervised liquid formula Procal (R-Kane)	– Measured weight and height.	**Distortion (actual shape):**<br>– M > W. Caucasians >African American	– Includes young people

	disturbance (as distortion, discrepancy and dissatisfaction) before and after weight loss.	– N= 82 overweight or obese (24 men + 58 women)<br>– Aged 18-60<br>– 4 weeks	given freely. 4 weeks of treatment. Mean weight loss of 6.4± 3 kg.	**Body image**(baseline + 4 weeks)**:**<br>– Figure Rating Scale (Stunkard) (actual for distortion, ideal for discrepancy)<br>– Dissatisfaction scale	– Initial > after weight loss (f=5.7)<br>– After weight loss: Early onset >adult onset (f=10.2)<br>**Discrepancy (ideal shape):**<br>– M > W. Caucasians > Hispanics<br>– Initial > after weight loss (f=32.3)<br>– After weight loss: Early onset >adult onset (f=8.5)<br>**Body image disturbance after weight loss**<br>– Decreased significantly for all. Early onset >adult onset.	– Small sample size.<br>– Diet is not a lifestyle, is just temporary.<br>– No exercise plan included.<br>– Patient perceptions were compared to counsellor perception, which is subjective.
**64**	Behavioural lifestyle weight-control program compared to a weight control with body image therapy	– USA<br>– N=65 (14 men +51 women)<br>– Aged 19-63<br>– BMI 27.5-60 (*measured)<br>– 1 year	2 randomized intervention (16 weeks):<br>o Weight control (LEARN = low fat). 1h/week dietitian<br>o Weight control + body image (basic calorie control + body image psychological therapy). 1h/week dietitian + 2h psychologist	Follow up: baseline, 16, 52 weeks.<br>– Measured weight.<br>– Adherence and satisfaction<br>**Body image:**<br>– Body Dysmorphic Disorder Examination—Self-Administration.<br>– Body Shape Questionnaire	**Weight loss**(No differences between groups at any point)<br>– 16 week: 8.91 kg; 52 week: 4.69 kg (53% weight loss retention).<br>**Body image in both groups:**<br>– Improved through time, from a severe to a normal range.<br>*Body Dysmorphic Disorder Examination—Self-Administration:*<br>– Normal range: Baseline: 25%; 16 week: 83%; 52 week: 70%.<br>– Baseline > 52 week (f=46.59). 16 week < 25 week (f=9.71)<br>*Body Shape Questionnaire:*<br>– Normal range: Baseline: 29%; 16 week: 86%; 52 week: 68%.<br>– Baseline > 52 week (f=43.69). 16 week < 25 week (f=16.13)<br>**Body image in weight control (without body image therapy):**<br>– Body image improvement through treatment correlated to greater weight loss (r=0.53) and weight loss retention (r=0.42).<br>**Body image dissatisfaction predictors:**<br>– Weight regain (r=0.29 and r=0.31)<br>– Low weight loss retention (r=0.56)	– Includes young people<br>– Small sample size.<br>– Intervention targeted behaviour changes that could help body image, not body image itself.
**65**	Evaluate a modified cognitive	– UK<br>– N=75 overweighe	Group intervention (10 week): healthy diet + physical activity + focus on:	Follow up: baseline, 6, 12 months.	**Weight loss:**<br>– After treatment: standard: 3.9 kg (t=5.92): modified: 1.3 kg	– Includes young people

	behavioural treatment for weight management without a focus on weight loss.	d women (38 standard+ 37 modified)<br>– Age: 18-65<br>– BMI >= 28 (*measured)<br>– 1 year	o Standard (control): energy restriction (1200 kcal)<br>o Modified (intervention): self-control + self-esteem. No focus on weight loss.	– Measured weight and height.<br>**Body image:**<br>– Body Satisfaction Scale.<br>– Body-Image Avoidance Questionnaire.<br>**Diet and physical activity**<br>– EPIC food frequency questionnaire<br>– Physical activity questionnaire<br>– Techumseh step test (Physical activity)	– After 1 year: standard: 3.6 kg (t=2.32): modified: 2.0 kg (t=2.41). (f=3.71)<br>– Significant differences (Standard > modified) at the end of the treatment, but not after 1 year.<br>**Diet and physical activity**<br>– No group by time interactions.Improvements through time:<br>– Diet: lower calorie (f=9.3), SFA, PUFA, MUFA and sucrose<br>– Physical activity: higher fitness (f=7.91) and reported activity.<br>**Body image:**<br>– No group by time interactions. Improvements through time:<br>– Body dissatisfaction (f=12.44)<br>– Body image avoidance (f=6.21)	– Small sample size.<br>– Biased to find the best of modified("predicted advantage of modified")
**18**	Excessive body preoccupation as an indicator of distortions in body image.	– Australia<br>– N=68 women<br>– Aged 18-65<br>– 15 weeks	– Weight reduction counselling courses at Macquarie University (slow lifestyle modification)<br>– Once a week: initial session + 10 clinical sessions (intensive counselling) + 4 measurement sessions (2nd6th11th15thcontact) where height, weight and skinfold measures were taken	**Body image**(2nd, 15thcontact)**:**<br>– Photograph of the subject in swimming costume. Images varied in the horizontal plane without distorting height.<br>– Identify: actual, ideal, goal.<br>– Random starting image and question	– ideal > goal > actual<br>– All saw themselves more obese than they were.<br>**Actual perceived body image**(% weight over perceived)**:**<br>– Baseline: Completers (19%) < drop-outs (26%) (t=7.8)<br>– Baseline: Low BMI (19%) < high BMI (24%) (t=5.03)<br>– Baseline (19%) > 15 week (8.8%) (real 8.9% less weight)<br>**Goal body image:**<br>– Reduction of 11.7% after treatment	– 1983<br>– Only women<br>– Includes young people<br>– Comparison according BMI

Abbreviations: BMI: Body Mass Index. UW: Underweight, NW: Normal weight, OW: Overweight, OB: Obese. M: Man, W: Women. y-o: years – old.
